# Signal Transducer and Activator of Transcription 3 (STAT3) Suppresses STAT1/Interferon Signaling Pathway and Inflammation in Senescent Preadipocytes

**DOI:** 10.3390/antiox10020334

**Published:** 2021-02-23

**Authors:** Aisha Y. Madani, Yasser Majeed, Houari B. Abdesselem, Maha V. Agha, Muneera Vakayil, Nour K. Al Sukhun, Najeeb M. Halabi, Pankaj Kumar, Shahina Hayat, Mohamed A. Elrayess, Arash Rafii, Karsten Suhre, Nayef A. Mazloum

**Affiliations:** 1 College of Health & Life Sciences, Hamad Bin Khalifa University (HBKU), Qatar Foundation, Doha 34110, Qatar; amadani@hbku.edu.qa (A.Y.M.); mvakayil@hbku.edu.qa (M.V.); 2 Department of Microbiology and Immunology, Weill Cornell Medicine-Qatar (WCM-Q), Qatar Foundation, Doha 24144, Qatar; yam2013@qatar-med.cornell.edu (Y.M.); nka4002@qatar-med.cornell.edu (N.K.A.S.); 3 Neurological Disorders Research Center, Qatar Biomedical Research Institute (QBRI), Hamad Bin Khalifa University (HBKU), Qatar Foundation, Doha 34110, Qatar; habdesselem@hbku.edu.qa; 4 Translational Research Institute, Academic Health System, Hamad Medical Corporation, Doha 3050, Qatar; magha@hamad.qa; 5 Department of Genetic Medicine, Weill Cornell Medicine-Qatar (WCM-Q), Qatar Foundation, Doha 24144, Qatar; nah2024@qatar-med.cornell.edu (N.M.H.); jat2021@qatar-med.cornell.edu (A.R.); 6 PatsnapI Pte Ltd., Singapore 209000, Singapore; pkumar@patsnap.com; 7 Department of Physiology and Biophysics, Weill Cornell Medicine-Qatar (WCM-Q), Qatar Foundation, Doha 24144, Qatar; shahina.hayat@hotmail.com (S.H.); kas2049@qatar-med.cornell.edu (K.S.); 8 Biomedical Research Center (BRC), Qatar University, Doha 2713, Qatar; m.elrayess@qu.edu.qa

**Keywords:** cellular senescence, inflammation, ROS, preadipocytes, SASP, type 2 diabetes

## Abstract

Obesity promotes premature aging and dysfunction of white adipose tissue (WAT) through the accumulation of cellular senescence. The senescent cells burden in WAT has been linked to inflammation, insulin-resistance (IR), and type 2 diabetes (T2D). There is limited knowledge about molecular mechanisms that sustain inflammation in obese states. Here, we describe a robust and physiologically relevant in vitro system to trigger senescence in mouse 3T3-L1 preadipocytes. By employing transcriptomics analyses, we discovered up-regulation of key pro-inflammatory molecules and activation of interferon/signal transducer and activator of transcription (STAT)1/3 signaling in senescent preadipocytes, and expression of downstream targets was induced in epididymal WAT of obese mice, and obese human adipose tissue. To test the relevance of STAT1/3 signaling to preadipocyte senescence, we used Clustered Regularly Interspaced Short Palindromic Repeats/CRISPR associated protein 9 (CRISPR/Cas9) technology to delete STAT1/3 and discovered that STAT1 promoted growth arrest and cooperated with cyclic Guanosine Monophosphate-Adenosine Monophosphate (GMP-AMP) synthase-stimulator of interferon genes (cGAS-STING) to drive the expression of interferon β (IFNβ), C-X-C motif chemokine ligand 10 (CXCL10), and interferon signaling-related genes. In contrast, we discovered that STAT3 was a negative regulator of STAT1/cGAS-STING signaling—it suppressed senescence and inflammation. These data provide insights into how STAT1/STAT3 signaling coordinates senescence and inflammation through functional interactions with the cGAS/STING pathway.

## 1. Introduction

Obesity is a major risk factor for type 2 diabetes (T2D) as well as an array of life-threatening cardiovascular diseases and obesity-related cancers. T2D is characterized by insulin resistance (IR)/hyperglycemia and is associated with dyslipidemia, hypertension, visceral obesity, glucose intolerance, and endothelial dysfunction [1,2,3]. The morbidity of these metabolic disorders constitutes a major health problem worldwide [1,2,3]. Obesity most likely occurs due to genetic, epigenetic, and lifestyle factors, such as reduced physical activity and increased calorie intake [1,2,3]. T2D and obesity adversely affect metabolic homeostasis over time and are associated with chronic inflammation and oxidative stress [4].

Recent evidence suggested that diet-induced obesity (DIO) in animals promoted premature aging and consequent white adipose tissue (WAT) dysfunction through the accumulation of cellular senescence [5,6]. This was accompanied by the activation of tumor suppressor p53, which was proposed to trigger inflammation response leading to IR and glucose intolerance [5,6]. Furthermore, it was proposed that excessive calorie intake led to the accumulation of oxidative stress in the adipose tissue of mice with T2D-like disease, which resulted in senescence-like features, such as positive staining with senescence-associated beta-galactosidase (SA-β-gal), increased expression of p53, and the cell cycle inhibitor p21^CDKN1A^, along with elevated levels of pro-inflammatory cytokines [5,6].

Cellular senescence is a state of irreversible growth arrest [7]. It occurs as a result of various cellular stress responses and has been proposed to drive aging-related degenerative disorders [8] and tumor progression [9]. Senescent cells are metabolically active irrespective of their inability to divide, and they are characterized by a senescence-associated secretory phenotype (SASP), in which hundreds of pro-inflammatory molecules, such as cytokines and chemokines among others, are secreted and believed to drive the adverse effects of inflammation in aging [10]. Persistent senescence in tissues is detrimental and can promote tissue dysfunction due to the secretion of SASP [11].

It is known that different fat depots make distinct contributions to the pro-inflammatory and clinical consequences of obesity [12]. Unlike the subcutaneous fat depot, the visceral fat depot is more strongly associated with obesity-linked inflammation and cytokine production [12]. The stromal vascular fraction of visceral fat is thought to be the major contributor to chronic inflammation in the obese state. This fraction of adipose tissue is comprised of pre-adipocytes, endothelial cells, immune cells, and other cell types [13,14]. Pre-adipocytes comprise 15–50% of the cells residing in WAT and constitute the biggest pool of progenitor cells in the body [12]. Though their main role is to differentiate into fat cells, their expression profile is different from fat cells and is more related to macrophages [15]. Pre-adipocytes express toll-like receptors and have the potential of full innate immune response [16,17]. Obesity or increased serial passage of pre-adipocytes was proposed to lead to the accumulation of senescent pre-adipocytes, which subsequently might initiate the infiltration and/or the activation of macrophages and other immune cells [12]. Upon activation, the WAT infiltrating macrophages participate in the inflammatory cycle by releasing even more chemokines and cytokines [12].

Components of SASP, including tumor necrosis factor-α (TNF-α) and interleukin 6 (IL-6), are increased in WAT and muscle tissues in obese humans and animal models and are thought to promote IR and T2D [18]. Emerging evidence has shown that the clearance of senescent cells in adipose tissue by pharmacogenetic approaches enhanced adipogenesis and metabolic function in obese animal models [19]. Furthermore, strategies to reduce the burden of senescent cells in these animals either by in vivo clearance or inhibiting SASP improved aging-related metabolic disorders, promoted insulin sensitivity, and reduced adipose tissue inflammation [20,21].

Cellular senescence can be induced via the activation of p53/21- and/or pRB/16-dependent pathways, which are thought to converge in promoting the senescence-associated growth arrest phenotype and in inducing a small subset of the pro-inflammatory SASP molecules [9]. Recent evidence has shown that the cytosolic DNA sensor cyclic Guanosine Monophosphate-Adenosine Monophosphate (GMP-AMP) synthase (cGAS) and its downstream effector, stimulator of interferon genes (STING), trigger inflammation during senescence [22]. The activation of this pathway in adipose tissue was caused by mitochondrial DNA damage that led to increased chronic inflammatory responses [23]. However, there is limited understanding of mechanisms that are engaged during obesity or subsequent oxidative stress-induced senescence in preadipocytes to promote senescence and inflammation.

In this study, we devised a physiologically relevant in vitro system and induced senescence in murine 3T3-L1 preadipocytes by repeated treatment with sublethal doses of hydrogen peroxide (H_2_O_2_) to investigate the underlying mechanisms triggering the development of the pro-inflammatory SASP. Importantly, the whole transcriptome analysis allowed us to discover disease-specific molecules involved in regulating SASP. Hence, targeting these molecules could be a therapeutic strategy for treating the inflammatory phenotype associated with senescence in obesity-driven T2D.

## 2. Materials and Methods

### 2.1. The 3T3-L1 Cell Culture and Senescence Induction Protocol

Mouse 3T3-L1 preadipocytes (#SP-L1-F, Zen-Bio, Durham, NC, USA) were maintained in Dulbecco’s modified Eagle medium (DMEM, catalogue no.12430054, Invitrogen, Carlsbad, CA, USA), supplemented with 10% calf serum (Catalogue no.16170078, Invitrogen) and 1% penicillin/streptomycin antibiotics (Catalogue no.15240002, Invitrogen). Human Embryonic Kidney 293 cells and contains the SV40 T-antigen (HEK293T) (ATCC) were used for viral packaging as described previously [24]. Cells were maintained in (DMEM) supplemented with 10% fetal bovine serum (FBS) (catalogue no. AlphaFBS-HI, Alphabioregen, Boston, MA, USA) and 1% penicillin/streptomycin antibiotics (catalogue no.15240002, Invitrogen). All cells were grown at 37 °C in a humidified incubator (5% CO2) and were sub-cultured at 70–80% confluence. 3T3-L1 preadipocytes were induced into senescence by two intermittent 3 h exposures to sublethal doses of H_2_O_2_ (Sigma, St. Louis, MO, USA) using the following protocol. Cells were plated at a density of 1.2 × 10^6^ in 10 cm dishes or 2.0 × 10^5^ in 6-well plates a day before the treatment (Day 1). The following day (Day 0), preadipocytes were treated with H_2_O_2_ (200 μΜ) for 3 h, followed by a phosphate-buffered saline (PBS) wash, and provided with fresh complete medium. On Day 3, cells were subjected to another 3-hr treatment with H_2_O_2_ (200 μΜ), washed once with PBS, and provided with fresh complete medium for an additional 2–4 days. The senescence phenotype was achieved by Day 5 and the samples were collected on different days post the first H_2_O_2_ treatment (0, 3, 5 and 7) for further analysis described below.

### 2.2. Mouse Experiments

The Institutional Animal Care and Use Committee (IACUC) at Weill Cornell Medicine-Qatar approved all the animal experiments (Protocol #2015-0026) and the project was carried out in an Association for Assessment and Accreditation of Laboratory Animal Care (AAALAC) International accredited facility. For the diet-induced obesity studies, 13-week old C57BL/6J mice were fed a high-fat diet (HFD) consisting of 60 Kcal% fat (Research Diets, Cat# D12492) or chow (Pico-Vac Lab Rodent Diet, Cat# 5061) for 20 weeks prior to sacrifice. To prepare the stromal vascular fraction (SVF), epididymal fat tissue was digested in a buffer containing 8 mg/mL Collagenase D [2511088866001, Roche, Basel, Switzerland] and 3 units/mL dispase II [25D4693, Sigma] for 30 min at 37 °C with gentle agitation on a shaker. The preparation was centrifuged and the pellet was washed 3 times with warm complete medium prior to re-suspension and plating in 6-well plates. To remove red blood cells (RBC), medium was gently removed after 3 h, followed by one PBS wash before medium replenishment. Cells were collected in Qiazol (#79306, Qiagen, Hilden, Germany) after overnight incubation and used for quantitative polymerase chain reaction (qPCR) analysis.

### 2.3. Experiments on Human Adipose Tissue Samples

Recruitment criteria of obese subjects undergoing weight reduction surgery were previously described [25]. Protocols were approved by the Institutional Review Board of Anti-Doping Lab Qatar (X2015000120) and were carried out in accordance with the Declaration of Helsinki as revised in 2008. Snap-frozen adipose tissue samples were homogenized in Qiazol and RNA was extracted using methods described in Section 2.7. qPCR analysis was performed in samples obtained from 23–24 subjects and the primer sequences are described in Table S1.

### 2.4. Senescence Associated β-Galactosidase (SA-β-Gal) Staining

The Senescence Associated β-Galactosidase (SA-β-gal) Staining Kit (catalogue no. 9860S, Cell Signaling, Leiden, The Netherlands) was used to detect senescent cells according to the manufacturer’s instructions. In brief, untreated and H_2_O_2_ treated preadipocytes at (Day 5 or Day 7) were washed twice in PBS and fixed with a fixative solution for 10 min at room temperature. After fixation, cells were washed twice with PBS, stained with a staining solution (pH 5.9–6.1) and incubated at 37 °C overnight. The stained preadipocytes were mounted with 70% glycerol and then imaged using a digital EVOS microscope from the Advanced Microscopy Group (AMG) for observing the formation of blue color staining in senescent preadipocytes. The number of SA-β-gal positive cells were quantified from four different microscopic fields, averaged and presented as a percentage.

### 2.5. Growth Curve

3T3-L1 preadipocytes were plated in 6-well plates at a density of 2.0 × 10^5^ cells/well. The numbers of the cells were recorded using TC10 automated cell counter (Bio-Rad, Hercules, CA, USA) at different time points post-plating and throughout H_2_O_2_ treatment.

### 2.6. Western Blotting

Preadipocytes were trypsinized, washed once in PBS, centrifuged, and cell pellets were snap-frozen on dry ice. Total protein was then extracted using RIPA buffer (Invitrogen) supplemented with protease inhibitor cocktail (Sigma), 10 mM sodium fluoride (Sigma), 1 mM sodium orthovanadate (Sigma), 1 mM Phenylmethylsulfonyl Fluoride (PMSF) (Sigma), 5 mM benzamidine (Sigma), 20 µg/mL calpain inhibitor (Sigma), 5 mM nicotinamide (Sigma), and 3 mM trichostatin A (InvivoGen). Cell lysates were homogenized by sonication (Sonic Raptor 250, OMNI International, Kennesaw, GA, USA) and centrifuged at 15,000× *g* for 10 min at 4 °C. Supernatants were collected for protein estimation using DC Protein Assay (Bio-Rad) with CLARIOstar microplate reader (BMG LABTECH, Offenburg, Germany). Fifty micrograms of protein samples were separated on sodium dodecyl sulfate polyacrylamide gel electrophoresis (SDS-PAGE) and transferred to a Polyvinylidene Fluoride (PVDF) membrane (Bio-Rad). The membranes were blocked with 4% bovine serum albumin (BSA) (Sigma) for an hour and probed overnight with the following primary antibodies: acetylated-p53 (Lys375) (Abcam, Cambridge, UK, Cat# ab61241, Research Resource Identifiers ID (RRID): AB_944590), tumor protein p53 (p53) (Abcam Cat# ab26, RRID: AB_303198), cyclin-dependent kinase inhibitor 1A (p21) (Abcam Cat# ab7960, RRID:AB_306174), C-X-C motif chemokine ligand 10 (CXCL-10) (Abcam Cat# ab8098, RRID:AB_306267), matrix metallopeptidase 13 (MMP13) (Abcam Cat# ab39012, RRID:AB_776416), matrix metallopeptidase 3 (MMP3) (Abcam Cat# ab53015, RRID:AB_881242), insulin-like growth factor binding protein 4 (IGFBP4) (Abcam Cat# ab77350, RRID:AB_1523812), interleukin 6 (IL6) (Abcam Cat# ab9324, RRID:AB_307175), tumor necrosis factor (TNFα) (Abcam Cat# ab1793, RRID:AB_302615), complement C3 (C3) (Abcam Cat# ab11887, RRID:AB_298669), ceruloplasmin (CP) (Abcam Cat# ab48614, RRID:AB_869113), dermatopontin (DPT) (Abcam Cat# ab118710, RRID:AB_10901710), signal transducer and activator of transcription 1 (STAT1) (Cell Signaling Technology Cat# 9172, RRID:AB_2198300), phospho STAT1 (Cell Signaling Technology, Cat# 9167, RRID:AB_561284), signal transducer and activator of transcription 3 (STAT3) (Cell Signaling Technology Cat# 9139, RRID:AB_331757), phospho STAT3 (Cell Signaling Technology, Cat#:9145, RRID:AB_2491009), cGAS (Cell Signaling Technology Cat# 31659, RRID:AB_2799008), STING (Cell Signaling Technology Cat# 13647, RRID:AB_2732796), interferon alpha (IFNα) (Thermo Fisher Scientific, Waltham, MA, USA, Cat# PA5-86767, RRID:AB_2803527), interferon beta (IFNβ) (Cell Signaling Technology Cat# 97450, RRID:AB_2800278) and β-actin (Cell Signaling Technology Cat# 3700, RRID:AB_2242334). The following day, these membranes were washed three times with tris-buffered saline tween (TBST) and incubated with the corresponding horseradish peroxidase (HRP) conjugated secondary antibodies including: Anti-rabbit Immunoglobulin G (IgG), Horseradish Peroxidase (HRP)-Linked Antibody (Cell Signaling Technology Cat# 7074, RRID:AB_2099233), anti-mouse IgG, HRP-linked antibody (Cell Signaling Technology Cat# 7076, RRID:AB_330924), anti-goat (Abcam Cat# ab6741, RRID:AB_955424) and anti-rat (Abcam Cat# ab6734, RRID:AB_955450). Signals were detected using SuperSignal West Dura Extended Duration Substrate (Thermo Fisher Scientific) using ChemiDoc^TM^ MP imaging system (Bio-Rad). Band intensities were quantified using ImageJ software https://imagej.nih.gov.

### 2.7. Quantitative Polymerase Chain Reaction (qPCR)

Total RNA was extracted using the miRNeasy Mini Kit (#217004, Qiagen) following the manufacturer’s instruction. To remove contaminating DNA, the sample was treated with DNase I (#79254, Qiagen) for 15 min at room temperature and subjected to RNA elution and purification as recommended. RNA was then quantified using NanoDrop (ThermoFisher Scientific) and reverse-transcribed to cDNA using a High Capacity RNA-to-cDNA Kit ((#4387406, Applied Biosystems, Foster City, CA, USA). Quantitative PCR (qPCR) was performed using SYBR Select Master Mix (Applied Biosystems) on a QuantStudio™ 6 Flex Real-Time PCR System (Applied Biosystems) using the following protocol: denaturation (50 °C, 20 s; 95 °C, 10 min); was followed by 40 amplification cycles (95 °C, 15 s; 60 °C, 1 min) and melting curve analysis steps (95 °C, 15 s; 60 °C, 1 min; 95 °C, 30 s; 60 °C, 15 s). The primers were designed using sequences spanning exon-exon junctions and the primer sequences are described in Table S1. RPLP0 was used as a housekeeping gene to normalize the expression of target genes used in our study.

### 2.8. Adipocyte Differentiation and Oil Red O Staining

Adipocyte differentiation of untreated (proliferating) and senescent 3T3-L1 preadipocytes was carried out as described previously [24]. In the case of untreated preadipocytes, cells were plated at 2.0 × 10^5^ cells in 6-well plate and differentiation was induced for 48 h 2 days after the cells reached 100% confluence. The differentiation cocktail contained 1 µM dexamethasone (Sigma), 0.5 µM 3-isobutyl-1-methylxanthine (Invitrogen) and 10 µg/mL insulin (Sigma) in DMEM medium containing 10% FBS and 1% penicillin/streptomycin antibiotics. Two days post-differentiation induction, the differentiation cocktail and medium were replaced by a fresh DMEM medium containing 10% FBS, 10 µg/mL insulin, and 1% penicillin/streptomycin antibiotics. The media were changed every two days until the end of the experiment. In parallel, senescent preadipocytes were subjected to the same differentiation protocol as proliferating preadipocytes on Day 7 of the senescence induction protocol.

Oil Red O staining (Sigma) of adipocytes was done as described [24]. Briefly, cells were washed twice with PBS and fixed with 4% paraformaldehyde at room temperature for 15 min. Fixed cells were then washed with PBS, rinsed with 60% isopropanol/PBS for 1 min and stained with Oil Red O for 1 h. Stained cells were washed with 60% isopropanol/PBS for 30 s and the excess stain was removed by washing with distilled water (3×). Nuclei were stained with hematoxylin stain (Invitrogen) for 8 min and washed with water (5×). Cells were mounted with glycerol (70%) glycerol and then imaged using a digital EVOS microscope from AMG. The number of positive Oil Red O stained cells were quantified from four different microscopic fields, averaged and presented as a percentage.

### 2.9. Transcriptomics and Ingenuity Pathway Analysis (IPA)

RNA-sequencing (RNA-seq) in combination with pathway analysis were performed on total RNA extracted from control (untreated) and senescent preadipocytes. The analysis was performed for experiments carried out at both 20% O_2_, as well as at 3% O_2_ and data were generated from three biological replicates. The RNA-seq cDNA library was sequenced at the Weill Cornell Medicine Qatar (WCMQ) Genomics Core Facility and read-mapping was performed at the WCMQ Bioinformatics Core Facility. The 100 bp paired reads were mapped to the mouse reference genome from GENCODE-built M10, GRCm38 assembly [26] with Tophat2 (version 2.1.0) [27] using Ensembl85 gene-annotation. Aligned reads were quantified with featureCounts function from Rsubread (version 1.22.3) [28] in the Bioconductor Package (version 3.3.2). All the read counts from conditions were combined into a data matrix based on gene-identifiers. Genes that had at least one read across conditions were selected and normalized the counts using the default method (relative log expression) in DESeq2 (version 1.12.4) [29]. Furthermore, differentially expressed genes between different conditions were identified using the Wald test from DESeq2. The differentially expressed genes were selected, and the significance threshold was set at a q-value of 0.05. Ingenuity Pathway Analysis software (IPA, Ingenuity Systems, http://www.ingenuity.com) was employed to identify signaling pathways that were differentially regulated in the two gene-sets. 

### 2.10. Dot Blotting

Senescent preadipocytes at Day 5 and control untreated cells were cultured in conditioned medium (serum-free), supplemented with ITS Premix Universal Culture Supplement (Corning) for 24 h. The conditioned medium was collected, centrifuged to remove cell debris and stored at –80 °C for further analysis. After normalizing the concentrations of the collected medium in each condition to the number of cells/well, 100 μL of the conditioned medium was applied to Bio-Dot SF Microfiltration Apparatus (Bio-rad) using nitrocellulose membrane. The membranes were blocked using 4% BSA in tris-buffered saline with 0.1% Tween ^®®^ 20 Detergent (TBST) for 1 h at room temperature. The membranes were then incubated with the primary antibody prepared at 1:1000 dilution in TBST supplemented with 2% BSA for 1 h at room temperature, washed with TBST (3×) for 5 min. After washing, membranes were incubated with the secondary antibody prepared at 1:10,000 dilution in 2% BSA/TBST for 1 h, and then washed with TBST (3×) for 5 min. Signals were detected in the presence of SuperSignal^TM^ West Dura Extended Duration Substrate (Thermo Fisher Scientific) using ChemiDoc^TM^ MP imaging system (Bio-Rad). Band intensities were quantified using ImageJ software https://imagej.nih.gov.

### 2.11. Generation of Clustered Regularly Interspaced Short Palindromic Repeats/CRISPR Associated Protein 9 (CRISPR/Cas9) Lentiviruses

Guide RNA lentiCRISPR v2 constructs targeting STAT1, STAT3, cGAS, STING and Non-targeting (NT) control were purchased from Genscript (Piscataway, NJ, USA) and generated as described previously [30]. The gRNA target sequences were: cGAS gRNA-1: AAACGGCTCTCGTCTTAGAT, STING gRNA-1: CGGCAGTTATTTCGAGACTC, STAT1 gRNA-1: GGTCGCAAACGAGACATCAT, STAT3 gRNA-1: CGATTACCTGCACTCGCTTC and NT control gRNA: GCTTTCACGGAGGTTCGACG. Briefly, in HEK293T cells the transfer plasmids were co-transfected with packaging plasmids using lipofectamine 2000 following manufacturer’s instructions. After 6 h, the medium was changed to DMEM supplemented with 10% FBS to improve virus stability. Lentiviral supernatants were harvested after 72 h, centrifuged at 1500 rpm at 4 °C for 5 min to pellet cell debris and filtered through a 0.45 µm membrane (Millipore, Burlington, MA, USA). Generated lentiviruses were used to infect 3T3-L1 preadipocytes using 4 μg/mL polybrene (Santacruz Biotechnology, Dallas, TX, USA) for 48 h and followed by 2 µg/mL puromycin selection (Life Technologies, Carlsbad, CA, USA). Infected cells were passaged (3×) in the presence of puromycin (2 µg/mL) prior to gene knock-out validation by Western blotting. Non Targeting (NT) and Knocked Out (KO) cells were maintained in DMEM medium supplemented with 10% calf serum, 2 µg/mL puromycin, and 1% penicillin/streptomycin antibiotics (Catalogue no.15240002, Invitrogen).

### 2.12. Data Analysis and Statistics

Experiments were performed at least three times independently, and results are presented as means ± standard error of the means (SEM). GraphPad Prism software (https://www.graphpad.com) was used to plot the data and perform statistical analysis using Student’s t-test for pairwise comparisons or ordinary one-way ANOVA (followed by post hoc analysis) for multiple parameter comparison. A *p*-value of less than 0.05 was considered statistically significant. 

## 3. Results

### 3.1. Induced Senescence in 3T3-L1 Preadipocytes with Repeated H_2_O_2_ Treatment Impairs Adipogenesis

**Figure 1:**
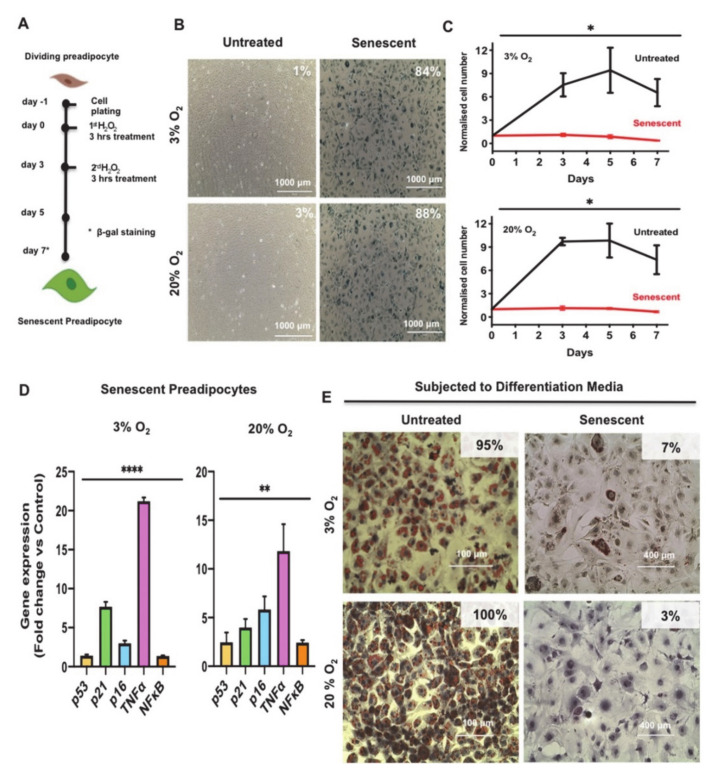
Induction of premature senescence in 3T3-L1 preadipocytes with H_2_O_2_ impairs adipogenesis. (**A**) Schematic representation of the H_2_O_2_ treatment protocol to induce premature senescence in 3T3-L1 preadipocytes. (**B**) Senescence-associated beta-galactosidase (SA-β-gal) staining of proliferating (untreated) or senescent preadipocytes grown at 3% O_2_ or 20% O_2_ as indicated. SA-β-gal positive cells were quantified from four different fields and represented as a percentage within the images. Scale bar, 1000 μm. (**C**) Growth curve analysis of 3T3-L1 preadipocytes subjected to the H_2_O_2_ treatment protocol as in A compared to control untreated cells. The number of senescent cells and untreated cells were counted on different day and represented as a fold-increase over the number of seeded preadipocytes on Day-1. Results are presented as means ± standard error of the mean (SEM) of three independent experiments. (**D**) Gene expression analysis for relevant markers of senescence and inflammation. Gene expression results (fold change vs. control) of each indicated marker are presented as means ± SEM of three independent experiments. (**E**) Oil Red O and hematoxylin staining images of untreated or senescent preadipocytes (Day 7) maintained at either 3% O_2_ or 20% O_2_ condition subjected to adipocyte differentiation protocol. Scale bars: 100 μm and 400 μm. Statistically significant difference: * *p* ≤ 0.05, ** *p* ≤ 0.01, **** *p* ≤ 0.0001 (Student’s t-test).

To investigate mechanisms that govern preadipocyte cellular senescence and WAT dysfunction in obesity, we employed murine 3T3-L1 preadipocytes as a cellular model and devised a senescence induction protocol by subjecting these cells to oxidative stress using sublethal concentrations of hydrogen peroxide (H_2_O_2_) as a source of reactive oxygen species (ROS). 3T3-L1 preadipocytes are fibroblast-like cells that can be differentiated into mature adipocytes and have been employed as a mouse in vitro model system in studying adipogenesis for decades [31]. Previously reported transcriptomics analysis suggested that adipocytes derived from 3T3-L1 preadipocytes closely resembled those derived from white adipose tissue, both in terms of gene-expression profile and mitochondrial bioenergetics [32]. Unlike human cells, murine cells such as the mouse embryo fibroblasts (MEF) exhibit increased sensitivity to oxidative stress and accumulate more DNA damage if cultured under the standard atmospheric oxygen-growth conditions (20% O_2_), which could interfere with cell proliferation and replicative senescence phenotypes [19]. Therefore, to rule out any confounding effects of higher oxygen growth condition on our experimental cellular senescence system, we cultured the 3T3-L1 preadipocytes at either atmospheric oxygen (20%) or in a hypoxia chamber set at lower oxygen concentration (3%), which mimics physiological oxygen concentration and then subjected these cells to an H_2_O_2_ treatment protocol to induce premature cellular senescence. A schematic representation of the time course and experimental conditions of the devised cellular senescence induction protocol are shown in Figure 1A.

Early passage 3T3-L1 preadipocytes were grown and maintained in culture at the respective oxygen concentration for two passages before initiating the senescence induction protocol. Cells were then seeded in 6-well plates at 2 × 10^5^ cells/well and then treated the next day (Day 0) with H_2_O_2_ (200 μM) for 3 h and similarly on the third day (Day 3). After each treatment, medium was replaced with normal medium and changed every other day until the conclusion of the experiment. Untreated control cells were grown in parallel at either 20% or 3% O_2_ and were split when the cells reached 70 % confluence. 

To monitor the levels of H_2_O_2_-induced senescence in 3T3-L1 preadipocytes subjected to the treatment protocol in Figure 1A, H_2_O_2_ treated and control cells were fixed and stained with a SA-β-gal staining kit to assay for the activity of senescence-associated β-gal (SA β-gal), a well-known biomarker of senescent cells [33]. As shown in Figure 1B, H_2_O_2_ treated preadipocytes showed a robust induction in percentage of SA β-gal expressing cells by day 7 as indicated (84% and 88% SA β-gal positive stained cells grown at 3% and 20% O_2_, respectively) compared to the untreated control preadipocytes (1% and 3% SA β-gal positive stained cells grown at 3% and 20% O_2_, respectively). Interestingly, the senescence feature of enlarged cells was observed at Days 5 and 7 post-treatment. Cells started to become enlarged and reached sizes of about 100 µm in diameter, which is 5–8 times larger than the untreated cells.

Cellular senescence is also characterized by permanent growth arrest [34]. To monitor changes in cellular growth in H_2_O_2_ treated vs. control cells, we performed growth curve analysis on these cells over a period of 7 days. Preadipocytes were treated with H_2_O_2_ (200 µM) according to our scheme shown in Figure 1A. Untreated control cells were grown in parallel at either 20% or 3% O_2_. Cell counts were recorded at the indicated time point (Days 0, 3, 5, and 7) under the respective O_2_ growth condition. Data in Figure 1C clearly show that H_2_O_2_ treatment resulted in arrested growth relative to the untreated regardless of the O_2_ growth condition.

It is known that activation of DNA damage response (DDR) and the signaling cascades via p53/p21 and/or pRB/p16 are critical players in promoting cellular senescence and growth arrest. Evidence has shown that the activation of these signaling cascades also regulate a small subset of proinflammatory genes, such as nuclear factor kappa B Subunit p65 (NFκB) and TNFα [35,36,37]. To assess the changes in these relevant markers of senescence after exposure to H_2_O_2_, we measured the expression levels of p53, p21, cyclin-dependent kinase inhibitor 2A (p16), TNFα, and NFκB in Day 7 senescent 3T3-L1 preadipocytes compared to untreated control. Gene expression analysis showed a significant increase in the levels of all these genes in senescent cells compared to controls at the two respective O_2_ growth conditions (Figure 1D).

Cellular senescence also impairs differentiation of preadipocytes [38]. To evaluate whether our protocol is suitable as a model for impaired adipogenesis, which is relevant to WAT dysfunction, we induced adipogenesis in control (untreated) and senescent preadipocytes using established protocols, followed by Oil Red O staining [24]. We observed a strikingly impaired adipogenesis differentiation response in senescent preadipocytes when compared to controls as indicated (7% and 3% Oil Red O positive stained cells grown at 3% and 20% O_2_, respectively) compared to the untreated control differentiated cells (95% and 100% Oil Red O positive stained cells grown at 3% and 20% O_2_, respectively) (Figure 1E). 

In summary, these experiments revealed that we have successfully devised a robust oxidative stress-induced senescence strategy in mouse preadipocytes. This allowed us to discover the altered molecular pathways and the underlying molecular mechanisms for cellular senescence and WAT dysfunction in obesity.

### 3.2. Transcriptomics and Enriched Pathway Analysis Uncover Critical Activated Pro-Inflammatory Pathways in Senescent Preadipocytes

**Figure 2:**
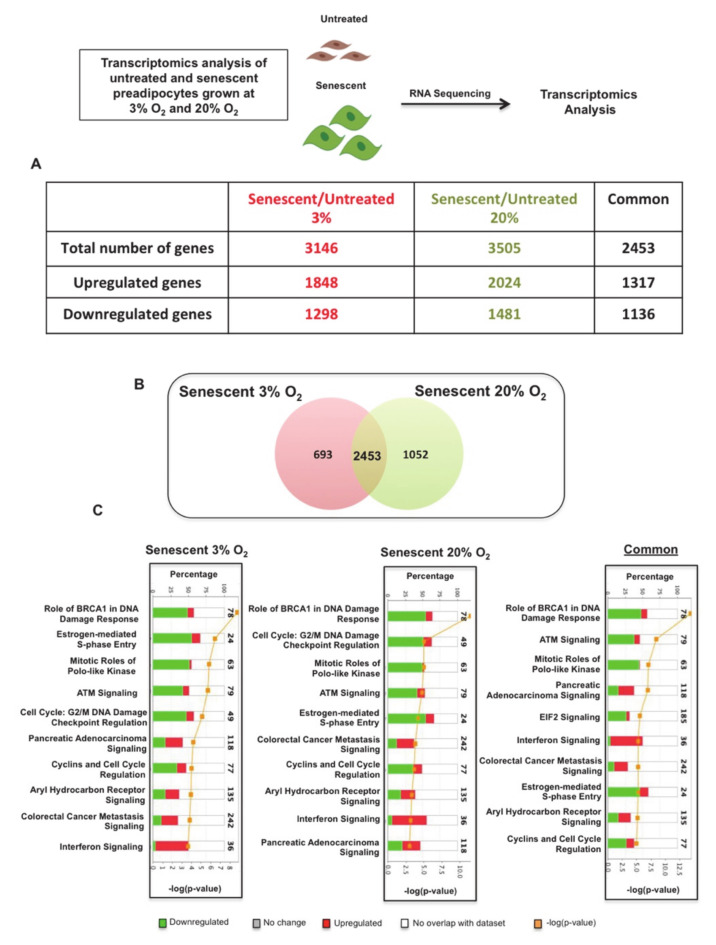
Transcriptomics analysis reveals common senescence signalling pathways in senescent preadipocytes grown at 3% O_2_ or atmospheric 20% O_2_ conditions. (**A**) List of the number of statistically significant transcriptomics changes in senescent preadipocytes grown at either 3% O_2_ or 20% O_2_ and the number of common genes between the two conditions compared to their respective untreated control. Highlighted in red and green are the numbers of up-regulated and down-regulated gene products respectively. (**B**) Venn diagram shows the overlap of genes between senescent preadipocytes cultured at 3% O_2_ and atmospheric O_2_. (**C**) 10 most significant canonical pathways that are altered in senescent preadipocytes grown at 3% O_2_, atmospheric O_2_, and common genes as indicated. Axes show percentages of overlapped genes in each pathway and *p*-value of overlap (Ingenuity Pathway Analysis (IPA) generated). Highlighted in red and green is the percentage of up-regulated and down-regulated genes products in each pathway, respectively.

To discover the pathways that sustain SASP in senescent preadipocytes, we performed whole transcriptome analysis (RNA-seq) on total RNA extracted from senescent preadipocytes and control cells (untreated) grown at either 3% O_2_ or atmospheric O_2_ concentrations (Figure 2). We discovered a total of 3146 and 3505 genes that were differentially expressed in senescent 3T3-L1 preadipocytes grown at either 3% or atmospheric O_2,_ respectively. We identified 1848 genes as up-regulated and 1298 as down-regulated in senescent preadipocytes grown at 3% O_2_. In senescent preadipocytes grown at atmospheric O_2_, 2024 genes were identified as up-regulated and 1481 were identified as down-regulated. 

To identify common molecular and cellular pathways that were altered in senescent preadipocytes grown under 3% or atmospheric O_2,_ we compared the two datasets and identified a total of 2453 common genes, of which 1317 were up-regulated and 1136 were down-regulated (Figure 2A,B). We subjected the total differentially regulated genes in these conditions along with common genes to IPA. Interestingly, the top ten most significant pathways were the same under the three analyses, which included DNA damage response pathways and cell-cycle regulatory pathways (Figure 2C). Our transcriptomics analysis helped us to identify differentially altered molecular pathways that were consistent with known and well-characterized growth arrest molecular pathways associated with cellular senescence. This analysis also highlighted a focused common set of senescence genes between the two oxygen growth conditions to consider for enrichment analysis to uncover critical and essential factors in driving senescence and sustaining SASP. 

**Figure 3:**
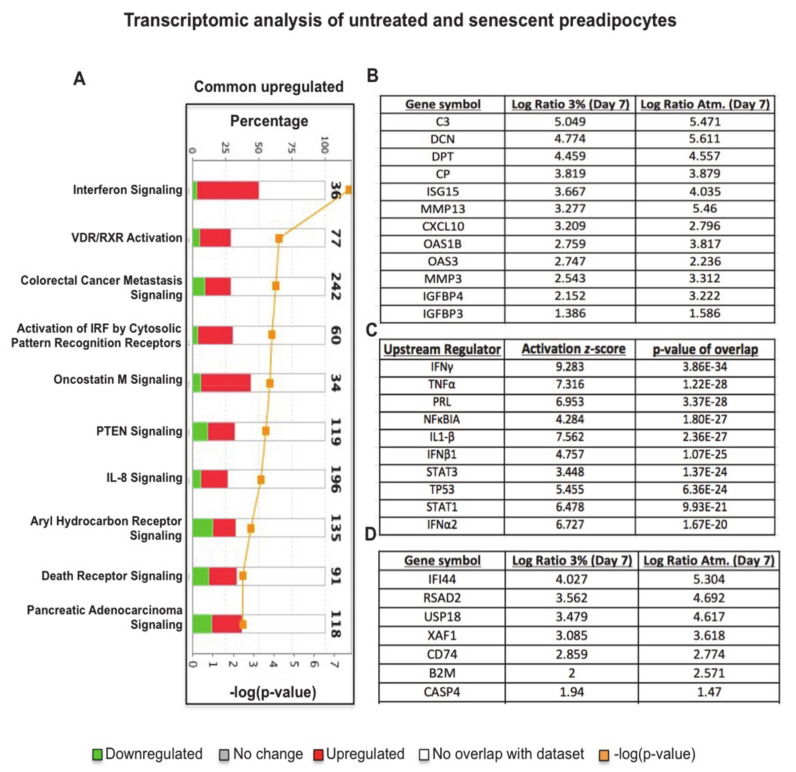
Enriched pathway analysis of up-regulated transcriptome changes in senescent preadipocytes reveal critical activated pro-inflammatory pathways. (**A**) Ten most significantly up-regulated canonical pathways of senescence transcriptome changes by IPA. Axes demonstrate percentages of overlapped genes in each pathway and *p*-value of overlap. Shaded in red and green is the percentage of up-regulated and down-regulated genes in each pathway. (**B**) Table listing the 12 most significantly up-regulated extracellular annotated genes (putative senescence-associated secretory phenotype (SASP) molecules) in dataset along with their expression values in the indicated condition. (**C**) Table shows IPA predictions of the 10 most significantly activated key regulators in senescent preadipocytes based on assigned IPA *p*-values of overlap and the degree of activation is based on IPA assigned z-scores of activation; z-score value above +2 signifies activated pathways. (**D**) Table listing the 7 most significantly up-regulated signal transducer and activator of transcription (STAT)1/3 targets along with their expression values in the indicated conditions.

To enrich our pathway analysis, we subjected the 1317 common up-regulated genes uncovered in our dataset to IPA and found the most significantly up-regulated canonical pathways, including Interferon signaling, Oncostatin M signaling and many other pro-inflammatory pathways (Figure 3A). This analysis also identified several putative SASP genes by selecting up-regulated genes that were annotated as extracellular molecules. Figure 3B lists the 12 most significantly up-regulated extracellular-annotated genes, including complement 3 (C3), decorin (DCN), dermatopontin (DPT), ceruloplasmin (CP), interferon-stimulated gene 15 (ISG15), matrix metallopeptidases (MMP13), C-X-C motif chemokine ligand (CXCL10), oligoadenylate synthetase (OAS1B), oligoadenylate synthetase (OAS3), matrix metallopeptidases (MMP3), insulin-like growth factor binding protein 4 (IGFBP4), and IGFBP3. This enriched analysis provided fascinating insights regarding the molecular mechanisms that promote pro-inflammatory SASP in senescent preadipocytes. By focusing our analysis on the most significant up-regulated common genes in senescent preadipocytes grown at 3% and atmospheric O_2_, we uncovered upstream key regulators using IPA software’s database of well-known pathway structures. We listed the ten most significantly activated upstream regulators, including interferon gamma (IFN-γ), TNF-α, prolactin (PRL), NFκB, interleukin 1 beta (IL1-β), IFN-β1, STAT3, TP53, STAT1, and IFN-α2 (Figure 3C). Transcriptomics analysis identified differentially expressed transcription factors and key regulators of inflammation along with several putative SASP-related molecules, including those belonging to the chemokine and matrix metalloproteinase (MMP) families.

In summary, the transcriptomics analysis followed by the enriched pathway analysis allowed us to identify critical transcription factors and activated pro-inflammatory pathways and to understand the molecular mechanism of senescent preadipocytes, which is relevant to obesity/T2DM disease states. 

### 3.3. Oxidative Stress Promotes Senescence-Associated Secretory Phenotype (SASP) Secretion, Interferon Signaling and Signal Transducer and Activator of Transcription 1 (STAT1) Expression in Senescent 3T3-L1 Preadipocytes

**Figure 4:**
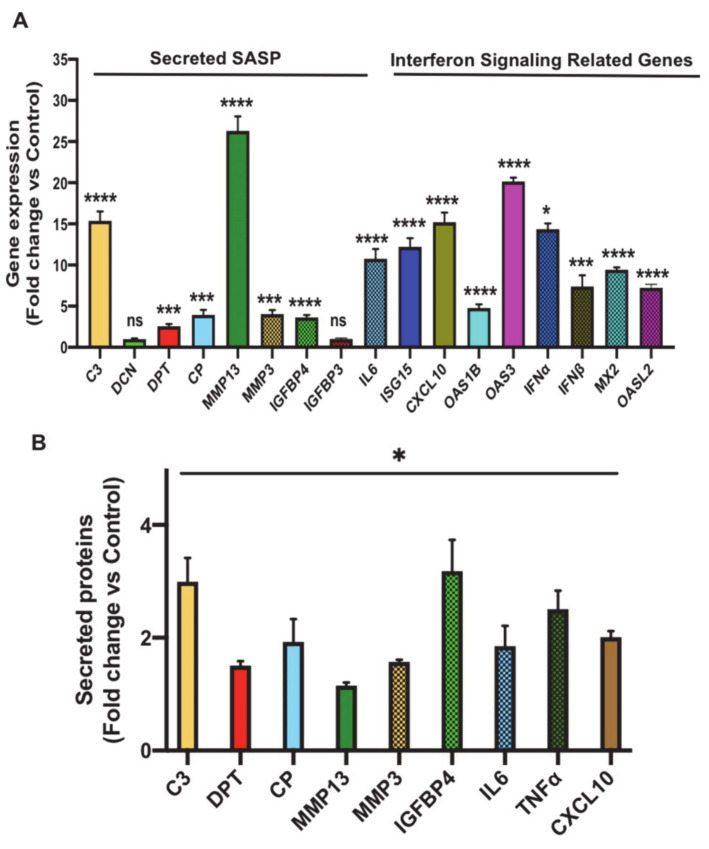
Validation of expression and/or secretion of the most differentially upregulated SASP and interferon/STAT signaling related genes that were identified in the transcriptomics analysis of senescent preadipocytes. (**A**) Gene expression analysis of the most significantly upregulated putative SASP molecules in senescent 3T3-L1 preadipocytes (Day 7) compared to untreated control by real-time quantitative polymerase chain reaction (qPCR). Results (fold change vs. control) are presented as means ± SEM from three independent experiments. * *p* ≤ 0.05, *** *p* ≤ 0.001, **** *p* ≤ 0.0001 (Student’s t-test). (**B**) Graph represents data for ImageJ quantification of the intensity of the signal of the indicated secreted molecule in conditioned media of senescent preadipocytes compared to untreated cells. Data are means ± SEM from 3 independent experiments. * *p* ≤ 0.05 (Student’s t-test).

To validate the transcriptomics data independently, we examined the expression and secretion profiles of the most significantly upregulated putative SASP genes in senescent 3T3-L1 preadipocytes compared to control cells. Shown in Figure 4A is the gene expression analysis of the tested genes and the data revealed a significant and robust induction in the expression of 10 out of 12 SASP-relevant genes, including C3 (*p* < 0.0001), DPT (*p* < 0.001), CP (*p* < 0.001), MMP13 (*p* < 0.0001), MMP3 (*p* < 0.001), IGFBP4 (*p* < 0.0001), ISG15 (*p* < 0.0001), CXCL10 (*p* < 0.0001), OAS1B (*p* < 0.0001), and OAS3 (*p* < 0.0001). We also examined interferon/STAT signaling by measuring the expression levels of the major inflammatory mediator IL6 and genes related to interferon signaling including IFNα, IFNβ, MX dynamin-like GTPase 2 (MX2) and oligoadenylate synthetase-like 2 (OASL2). Expression of these molecules was robustly induced.

To assess the secretion profile of these molecules during senescence, we collected conditioned media from Day 5 senescent preadipocytes and control (untreated) cells and compared the secretion levels of the aforementioned validated set of SASP molecules using Dot-Blot experiments as described in Materials and Methods. The Dot-Blot experiments revealed a significant increase in the secretion level of all the tested SASP molecules (Figure 4B and Figure S1). A subset of 4 molecules whose fold secretion level ≥ 2 induction was chosen for further characterization and this included C3, CP, IGFBP4, CXCL10. In summary, the above experiments served to independently validate the transcriptomics analysis.

### 3.4. Oxidative Stress Activates STAT1 and STAT3 and Promotes Induction of Their Targets

**Figure 5:**
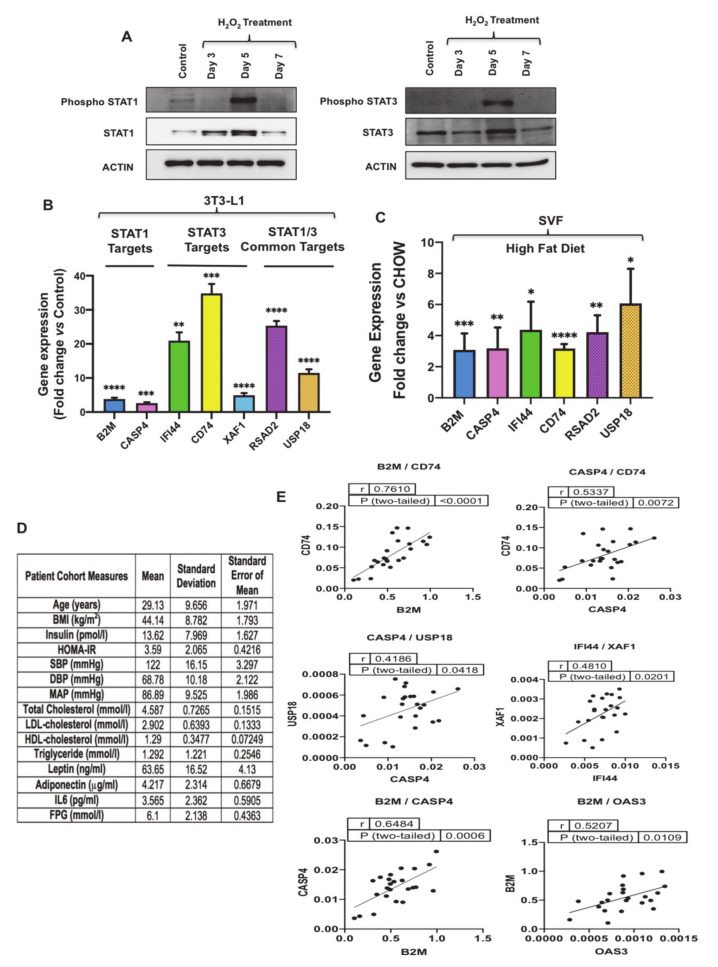
STAT1 and STAT3 activation in senescent preadipocytes and gene expression validation of their targets in senescent 3T3-L1, stromal vascular fraction (SVF) and human adipose fat tissues. (**A**) Western blot analysis of phospho-STAT1 (Y701), STAT1, phospho-STAT3 (Y705), STAT3 in proliferating and H_2_O_2_ treated preadipocytes at different time points. Actin was used as a loading control. (**B**) Gene expression analysis of the most significantly upregulated STAT1/3 targets in senescent preadipocytes by real-time qPCR. Results (fold change vs. control) are presented as means ± SEM from three independent experiments. ** *p* ≤ 0.01, *** *p* ≤ 0.001, **** *p* ≤ 0.0001 (Student’s t-test). (**C**) Gene expression analysis of STAT1/3 targets in SVF of high-fat diet mice by real-time qPCR. Gene expression results (fold change) are presented as means ± SEM from three independent experiments. * *p* ≤ 0.05, ** *p* ≤ 0.01, *** *p* ≤ 0.001, **** *p* ≤ 0.0001 (Student’s t-test). (**D**) Table of patient cohort measures selected for the study. Body Mass Index (BMI), Homeostatic Model Assessment for Insulin Resistance (HOMA-IR), Systolic Blood Pressure (SBP), Diastolic Blood Pressure (DBP), Mean Arterial Pressure (MAP), Low Density Lipoprotein (LDL), High-Density Lipoprotein (HDL). (**E**) Correlation analysis of STAT1/3 targets gene expression data in human adipose tissue.

To evaluate the expression of the top predicted activated proinflammatory transcription factors in the dataset, including STAT1 and STAT3 (activation z-score 6.478 and 3.448 respectively), we measured the level of their phosphorylation in senescent preadipocytes. Upon JAK activation, STAT1 and STAT3 become phosphorylated on their tyrosine residues (Y701) and (Y705), respectively [39]. Therefore, to monitor the activity of STAT1 and STAT3 in senescent preadipocytes, we measured the phosphorylation levels of Y701 (STAT1) and Y705 (STAT3), along with the total levels of STAT1 and STAT3 by Western blot analysis performed on lysates extracted from proliferating (untreated) and treated 3T3-L1 preadipocytes on Days 3, 5 and 7 (Figure 5A and Figure S2). The data revealed that phosphorylation of STAT1 and STAT3 at the indicated tyrosine residues was significantly increased at Day 5 of H_2_O_2_ treatment and the induction of total protein for both proteins was observed at Day 5 as well. We also validated the expression of STAT1 and STAT3 targets identified from transcriptomics data in Figure 3D, which included molecules exclusive for STAT1 including beta-2-microglobulin (B2M), caspase 4 (CASP4), STAT3 including interferon-induced protein 44 (IFI44), CD74, and XIAP associated factor 1 (XAF1), or common targets for STAT1/3 including radical S-adenosyl methionine domain containing 2 (RSAD2) and ubiquitin-specific peptidase 18 (USP18). Shown in Figure 5B is the gene expression analysis of STAT1/3 targets and the data revealed a robust induction in the expression of these genes in senescent 3T3-L1 preadipocytes. These targets were investigated further in Stromal Vascular Fraction (SVF) isolated from epididymal WAT of mice fed chow or high-fat diet (to promote obesity and WAT dysfunction/inflammation) (Figure 5C). Gene expression analysis revealed a significant induction in the expression of transcriptional targets of STAT1/3. These results are qualitatively similar to our discovery in senescent 3T3-L1 preadipocytes; thereby highlighting that activation of STAT1/3 in preadipocytes in obese WAT may promote inflammation.

To investigate relevance of STAT1 and STAT3 signaling to human obesity, expression analysis of STAT1/3 transcriptional targets was performed in an existing cDNA library prepared from adipose tissue samples obtained from obese human subjects. We analyzed 24 samples, and mean age of the subjects was 29.13 years and mean body mass index (BMI) was 44.14 (Figure 5D). Interestingly, correlation analysis of gene expression revealed significant positive correlation between key target genes including B2M/OAS3, B2M/CD74, B2M/CASP4, CASP4/USP18, CASP4/CD74 and IFI44/XAF1 regulated by STAT1 and STAT3 (Figure 5E).

In summary, the data described here show that STAT1 and STAT3 phosphorylation are induced in senescent 3T3-L1 and the expression of their downstream targets was robustly induced in senescent 3T3-L1 preadipocytes, SVF isolated from epididymal WAT of obese mice, and obese human adipose tissue.

### 3.5. GMP-AMP Synthase-Stimulator of Interferon Genes (cGAS-STING) Pathway Regulates C-X-C Motif Chemokine Ligand 10 (CXCL10) and Type I Interferon Effector Molecules in Preadipocytes

**Figure 6:**
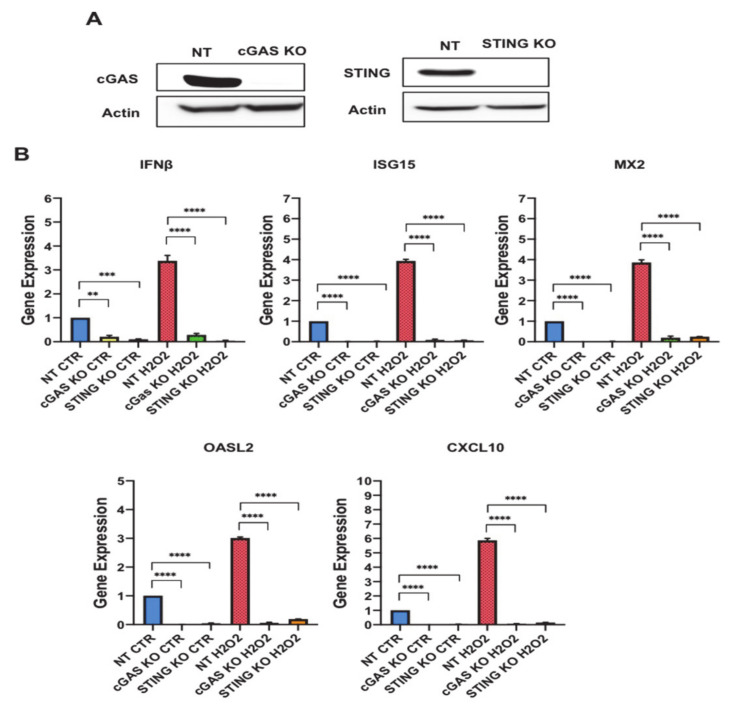
GMP-AMP synthase (cGAS) and stimulator of interferon genes (STING) regulate the gene expression of interferon signalling-related genes in preadipocytes. (**A**) Western blot assessing the protein levels of cGAS and STING in control Non Targeting (NT), cGAS Knockout (KO), STING KO cells. Actin was used as loading control. (**B**) Gene expression analysis for interferon signalling-related genes (interferon β (IFNβ), interferon-stimulated gene 15 (ISG15), MX dynamin-like GTPase 2 (MX2), oligoadenylate synthetase-like 2 (OASL2) and C-X-C motif chemokine ligand 10 (CXCL10)) in untreated NT, cGAS KO, and STING KO preadipocytes vs. H_2_O_2_ treated counterparts as indicated. Results (Relative expression) are presented as means ±SEM from three independent experiments. ** *p* ≤ 0.01, *** *p* ≤ 0.001, **** *p* ≤ 0.0001 (ANOVA with post hoc Tukey test).

CXCL10 and upstream regulators are members of interferon signaling cascade and are believed to participate in SASP. The robust induction observed in the secretion of SASP molecules has been shown to be stimulated by cytosolic DNA-sensing pathway GMP-AMP synthase-stimulator of interferon genes (cGAS-STING) [22]. To investigate the involvement of cGAS-STING signaling in regulating SASP secretion in senescent preadipocytes, we generated cGAS (cGAS KO) and STING (STING KO) knockout preadipocytes using CRISPR/Cas9 technology. To control for lentiviral infection, we used a lentiviral NT construct and employed it as a control for the downstream functional characterization studies. Using Western blot analysis, we validated gene deletion and demonstrated undetectable protein levels of cGAS and STING in the respective KO cells (Figure 6A). 

To evaluate the relevance of the cGAS-STING DNA sensing system to the senescence secretory phenotype in preadipocytes, we treated the KO preadipocytes with H_2_O_2_ following our established protocol. Afterward, gene expression analyses for both knockout preadipocytes were performed to monitor changes in CXCL10 and interferon signaling related genes including (IFNβ, ISG15, MX2, OASL2 and CXCL10). All these genes were significantly upregulated in the NT senescent preadipocytes (IFNβ: 4 fold (*p* < 0.0001), ISG15: 4 fold (*p* < 0.0001), MX2: 4 fold (*p* < 0.0001), OASL2: 3 fold (*p* < 0.0001), and CXCL10: 6 fold (*p* < 0.0001)). cGAS KO and STING KO preadipocytes exhibited a significant reduction in the mRNA levels of these genes compared to NT in untreated preadipocytes, and the H_2_O_2_ treatment did not alter the expression of these genes (Figure 6B and Figure S3). Therefore, these results indicated that the cGAS-STING pathway regulates CXCL10 and interferon signaling related genes in preadipocytes and predicts the involvement of predominant transcription factors impacting on cGAS-STING signaling to regulate SASP secretion.

### 3.6. STAT1 and STAT3 Exhibit Opposing Functions in Regulating Senescence-Associated Growth Arrest Phenotype

**Figure 7:**
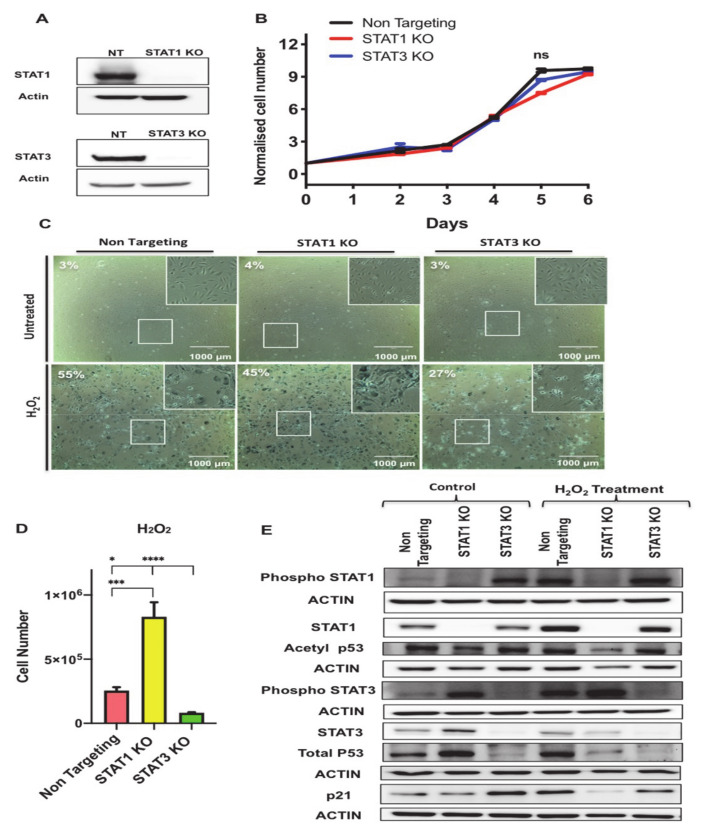
Antagonistic functions of STAT1 and STAT3 in regulating growth arrest and cell survival phenotypes in senescent preadipocytes. (**A**) Western blot assessing the protein levels of STAT1 and STAT3 in control NT, STAT1 KO, STAT3 KO cells. Actin was used as loading control. (**B**) Growth curve analysis of untreated non-targeting (NT), STAT1 KO, and STAT3 KO preadipocytes. The number of live preadipocytes in each condition was counted on different days and represented as a fold-increase over the number of seeded preadipocytes on Day-1. Results are presented as means ± SEM of three independent experiments. (**C**) Senescence-associated β-galactosidase (SA-β-gal) staining of proliferating (untreated) or H_2_O_2_ treated non-targeting (NT), STAT1 KO, and STAT3 KO preadipocytes. SA-β-gal positive preadipocytes were quantified from four different fields and represented as a percentage as indicated within the images. Scale bar: 1000 μm. (**D**) Graph represents the count of cell number of NT, STAT1 KO, and STAT3 KO following H_2_O_2_ treatment protocol. Results are presented as means ±SEM of three independent experiments. * *p* ≤ 0.05, *** *p* ≤ 0.001, **** *p* ≤ 0.0001 (ANOVA with post-hoc Tukey test). (**E**) Western blot of phospho-STAT1 (Y701), STAT1, phospho-STAT3 (Y705), STAT3, acetylated p53 (Lys375), total p53, p21 in untreated NT, STAT1 KO, and STAT3 KO preadipocytes vs. H_2_O_2_ treated counterparts as indicated. Actin was used as loading control. Results are represented as means ± SEM from three independent experiments.

To investigate the functions of STAT1 and STAT3 in regulating growth arrest in senescent 3T3-L1 preadipocytes, we knocked out STAT1 and STAT3 genes in these preadipocytes using CRISPR-Cas9 technology as described in the Material and Methods section and used lentiviral NT construct as a control. STAT1 and STAT3 protein levels were undetectable by western blotting in protein lysates extracted from the respective knockout cells (Figure 7A).

First, we examined the effect of STAT1 and STAT3 deletion on cellular growth in 3T3-L1 preadipocytes. We performed growth curve analysis of NT, STAT1 KO and STAT3 KO preadipocytes and counted the number of cells on different days (0, 2, 3, 4, 5, and 6) post-seeding. Data presented in Figure 7B shows that there was no significant difference observed on cellular growth phenotype in STAT1 KO and STAT3 KO compared to NT control cells.

To examine the function of STAT1 and STAT3 in oxidative-stress induced senescence in 3T3-L1 preadipocytes, we subjected NT, STAT1 KO and STAT3 KO preadipocytes to the H_2_O_2_ treatment protocol as described in Figure 1A and measured the activity of the SA-β-gal biomarker. Untreated NT, STAT1 KO and STAT3 KO preadipocytes and Day 7 post H_2_O_2_-treated counterparts were fixed and stained for SA β-gal as shown in (Figure 7C). The percentage of SA-β-gal expressing cells of the control untreated NT, STAT1 KO, and STAT3 were recorded as 3%, 4%, and 3%, respectively, and those of the treated counterparts were 55%, 45%, and 27%, respectively. Interestingly, counts of the number of total live cells 7 days post H_2_O_2_ treatment revealed a significant increase in the number of surviving cells in STAT1 KO compared to NT and STAT3 KO. The cell number of H_2_O_2_-treated STAT1 KO preadipocytes was three times higher than that of the H_2_O_2_-treated NT and six times higher that of the H_2_O_2_-treated STAT3 KO preadipocytes (Figure 7D) indicating that STAT1 and STAT3 act antagonistically in regulating the growth arrest phenotype under oxidative stress-induced senescence conditions.

To assess changes in some of the molecular markers of growth arrest upon deleting either STAT1 or STAT3, we measured the levels of acetylated p53 (Lys375) and the total protein levels of p53 and p21 by Western blot analysis performed on lysates extracted from untreated and H_2_O_2_-treated NT, STAT1 KO and STAT3 KO preadipocytes (Figure 7E and Figure S4). It has been reported in many cell types that p53 acetylation increases p53 protein stability and p53-dependent activation of apoptosis and senescence [40]. Importantly, DNA damage mediated acetylation of p53 at Lys381 has been known to prevent p53 ubiquitination degradation [41]. Therefore, investigating the changes in acetyl p53 will provide direct affects of its transcriptional activity and cell fate. Our data revealed a significant reduction in the acetylated form of p53 (Lys375) in STAT1 KO compared to NT and STAT3 KO in both the untreated and H_2_O_2_ treated conditions. There was no significant difference in the levels of the acetylated form of p53 (Lys375) in STAT3 KO compared to NT. Furthermore, the total protein levels of p53 were increased in untreated STAT1 KO preadipocytes compared to untreated NT and STAT3 KO, respectively. However, upon treatment with H_2_O_2_ p53 protein levels were reduced. Strikingly, the protein levels of p53 were decreased in both untreated and treated condition of STAT3 KO compared to NT control. However, the p21 protein levels were reduced in H_2_O_2_ treated STAT1 KO three fold (*p* < 0.0001) and two fold (*p* < 0.0001) compared to treated NT and STAT3 KO respectively and were increased by about 3 fold (*p* < 0.0001) in untreated STAT3 KO compared to untreated NT control. 

We also measured the level of phosphorylation of STAT1 (Y701) and STAT3 (Y705) to assess the changes in their activity after deleting STAT1 or STAT3. It has been reported that phospho-STAT3 Tyr705, and phospho-STAT1 Tyr701 are involved in modulating inflammatory responses in different diseases [42,43]. Interestingly, our data show STAT3 phosphorylation was increased in untreated STAT1 KO cells when compared to NT control (*p* < 0.01).

Therefore, the data described here clearly show opposing functions of STAT1 and STAT3 in regulating the growth arrest phenotype during senescence, which may be explained by their differential regulation of p53/p21 signaling.

### 3.7. STAT3 Aantagonizes STAT1 Regulation of STING and Downstream Molecules

**Figure 8:**
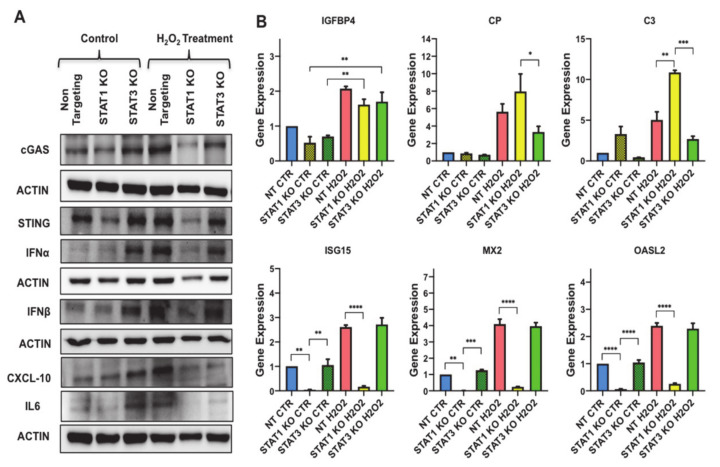
STAT1 functionally interacts with cGAS/STING to drive the expression of CXCL10 and antiviral response genes and STAT3 negatively regulates this interaction. (**A**) Western blot assessing the protein levels of cGAS, STING, IFNα, IFNβ, CXCL10 and IL6 in untreated NT, STAT1 KO, and STAT3 KO preadipocytes vs. H_2_O_2_ treated counterparts as indicated. Actin was used as loading control. Results are represented as means ± SEM from three independent experiments. (**B**) Gene expression analysis of most significantly upregulated SASP molecules in untreated NT, STAT1 KO, and STAT3 KO preadipocytes vs. H_2_O_2_ treated counterparts as indicated including IGFBP4, CP, and C3 along with interferon signalling-related genes (ISG15, MX2 and OASL2). Results (Relative expression) are presented as means ± SEM from three independent experiments. * *p* ≤ 0.05, ** *p* ≤ 0.01, *** *p* ≤ 0.001, **** *p* ≤ 0.0001 (ANOVA with post hoc Tukey test).

The results presented above prompted us to test whether STAT1 and STAT3 could impact on cGAS-STING signaling to regulate inflammatory phenotype associated with senescence. To uncover the role of cGAS-STING sensing machinery in STAT1 KO and STAT3 KO preadipocytes, we evaluated the protein levels of cGAS and STING in these cells (Figure 8A and Figure S5A). Surprisingly, our data revealed a significant reduction in the levels of STING protein in STAT1 KO control preadipocytes with no significant change in cGAS protein compared to STAT3 KO control preadipocytes. This reduction was promoted further upon subjecting the preadipocytes to H_2_O_2_-induced senescence protocol, indicating the critical function of STAT1 in regulating the expression of STING. However, STAT3 deletion was associated with increased cGAS and STING expression in control preadipocytes, and this expression was reduced when they were subjected to H_2_O_2_-induced senescence protocol.

Next, we measured the protein levels of SASP that are secreted in response to cGAS-STING signaling in STAT1 KO and STAT3 KO preadipocytes including IFNα, IFNβ, and CXCL10 and the major inflammatory mediator IL6 by Western blotting (Figure 8B and Figure S5B). Surprisingly, STAT3 deletion in control preadipocytes was accompanied by an increase in the protein levels of IFNα (~10 fold *p* < 0.001)), IFNβ (~3 fold *p* < 0.05)) and CXCL10 (~4 fold *p* < 0.001), and IL6 (~4 fold *p* < 0.01) implying the existence of a robust inflammatory response in these knockout preadipocytes. The protein levels of IFNα, IFNβ, CXCL10 and IL6 were reduced in H_2_O_2_-treated STAT3 KO preadipocytes compared to H_2_O_2_-treated NT. On the other hand, STAT1 KO preadipocytes showed a non-significant change in the protein levels of all these SASP-relevant genes compared to non-targeting; however when compared to STAT3 KO preadipocytes, it revealed a significant reduction in the protein level of all these SASP-relevant genes.

We also investigated the role of STAT1 and STAT3 in regulating senescence-associated inflammation by monitoring the changes SASP molecules including IGFBP4, CP and C3 in Figure 6B. Using qPCR experiments, we demonstrated a significant induction in the expression of IGFBP4 (~2 fold *p* < 0.01), CP (~7 fold *p* < 0.05), and C3 (~18 fold *p* < 0.05) in NT senescent preadipocytes compared to control. H_2_O_2_-treated STAT3 KO preadipocytes showed a 50% reduction in the expression of CP and C3 versus H_2_O_2_-treated NT. The H_2_O_2_-treated STAT1 KO preadipocytes showed a robust induction in the expression of CP (~8 fold *p* < 0.01) and C3 (~10 fold *p* < 0.001) compared to NT preadipocytes. Interestingly, STAT1 KO preadipocytes showed a significant reduction in the expression of CXCL10 when compared to H_2_O_2_-treated NT. 

We also performed gene expression analysis to monitor the changes in interferon signaling related genes (e.g., ISG15, MX2, OASL2) as shown in Figure 8B. The expression of these genes in H_2_O_2_-treated STAT3 KO preadipocytes was similar to NT senescent preadipocytes. However, the data revealed a significant reduction in the mRNA levels of these genes (ISG15, MX2 and OASL2) in STAT1 KO preadipocytes compared to NT and STAT3 KO preadipocytes, denoting the importance of STAT1 in regulating the expression of these molecules in senescent preadipocytes. In summary, these findings demonstrated an inverse function of STAT1 and STAT3 in regulating cGAS-STING signaling and the downstream interferon signaling-related genes in oxidative stress.

## 4. Discussion

Numerous studies have been published in recent years showing that inflammation is a major pathogenic mediator for the development of IR induced by obesity [5,44]. Obesity has been suggested to accelerate adipose tissue aging via increased cellular senescence in the WAT and the activation of p53/p21 axis [5,45]. In addition, dramatic increase in the infiltration of macrophages and other immune cells has been reported in WAT tissues in obese animal models and in tissues collected from obese subjects [46]. Furthermore, the levels of the pro-inflammatory cytokines TNF-α and IL-6 are elevated in WAT and muscle tissues of obese human and mouse models and cause IR and T2DM [5,47]. Although SASP is an important feature of senescent cells, little is known regarding the mechanistic processes that drive the senescence-associated growth arrest phenotype and associated secretion of pro-inflammatory SASP molecules in obesity, how it contributes to T2DM progression, and whether targeting these mechanism could be a beneficial therapeutic approach.

In this study, we have addressed these gaps by establishing an in vitro model system to induce senescence in preadipocytes and performing transcriptomics and pathway analyses to gain insight into pathways that drive senescence and inflammation. Our transcriptomics analyses uniquely reported the common genes in senescent cells grown at 3% and 20% O2 conditions, thus ruling out off-target effects of high O_2_ levels that could influence the study outcome. Several previous studies have conducted transcriptomics analyses as a powerful tool to study adipogenesis in 3T3L1 or to identify potential therapeutic senolytic drugs [48,49]. This study evaluated the senescence- associated secretory profile that accompanied senescence in 3T3L1 preadipocytes. We observed a significant induction in the levels of C3, CP enzyme, growth factor IGFBP4, and cytokine CXCL10 and interferon signaling-related genes including (IFNβ, ISG15, MX2 and OASL2), some of which have been recognized for their role either in obesity or inflammation. High serum C3 and plasma CP enzyme levels have been associated with obesity [50,51]. These studies suggest that the identified SASP components are expected to exert various effects or paracrine activities on senescent cells, which could be either harmful or beneficial. Thus, evaluation of the secretory profile extends the recent findings on SASP, especially in senescent preadipocytes. 

We further validated the top upregulated transcription factors, besides P53 and NFκB, by bioinformatics and pathway analyses that included STAT1 and STAT3. Our data revealed that STAT1/3 were robustly phosphorylated in senescent preadipocytes, and their transcription targets were significantly induced in senescent preadipocytes, SVF derived from HFD mice, and human adipose tissue. It is known that STATs are classically activated post-translationally by Janus kinase (JAK)-dependent phosphorylation of a tyrosine residue in the carboxy-terminus [52]. STAT-target genes regulate diverse biological functions including immune response, cellular growth, differentiation, and energy expenditure [53]. Evidence for cytokine-induced senescence and functional roles for JAK/STAT signaling in promoting some SASP molecules is emerging [54]. Therefore, these observations led us to study the biological signals coordinated by STAT1 and STAT3 in driving senescence and promoting inflammation and insulin resistance in preadipocytes. 

Interestingly, our analysis also unveils the differentially expressed transcriptome in senescent preadipocytes, where the most significantly up-regulated canonical pathway is interferon signaling. Several recent reports have shown that interferon signaling can be activated in response to SASP induction following the initiation of cGAS-STING signaling [55]. In brief, following a response to DNA damage, cytosolic self-DNA escapes into the cytosol where it is recognized by the cGAS-STING pathway, which then stimulates the production of interferons and inflammatory factors [55]. The SASPs activated by interferon signaling following the activation of cGAS-STING include CXCL10 and genes that play a critical role in interferon signaling, including IFNβ, ISG15, MX2, and OASL2 [56,57,58,59,60]. In our study, we evaluated the mRNA expression of these genes in oxidative stress conditions in preadipocytes in the absence of cGAS and STING. We reported that CXCL10 and interferon signaling-related genes were significantly down regulated in these conditions, which indicated that they are regulated by the cGAS-STING signaling in senescent preadipocytes. However, several studies have reported a different role for interferon-stimulated genes in their response to cGAS signaling [61,62]. For instance, Ghosh et al. reported a distinct regulation by OASL2 through inhibiting the activity of cGAS to limit interferon induction [61]. 

Next, we performed knockout experiments using CRISPR-Cas9-mediated deletion of STAT1 and STAT3. Notably, our data demonstrated distinctive effects when compared to their phenotype in oxidative stress-induced senescence. STAT1 deletion in preadipocytes showed reduced sensitivity to senescence growth arrest characterized by the reduction of acetylated p53 and p21, whereas deletion of STAT3 reduced the survival of senescent preadipocytes. Interestingly, we demonstrated an induction in the levels of both the phosphorylated and total protein forms of STAT3 following STAT1 deletion in control preadipocytes. On the other hand, phospho-STAT1 levels were increased upon the deletion of STAT3 levels. We also showed that STAT3 deletion resulted in an induction of acetylated p53 and promoted the activation of p21, furthering cell growth arrest. We speculate that during oxidative stress, the activation of STAT1 inhibits the downregulation of the p53/p21 axis governed by STAT3 through suppressing STAT3 function. A recent study by [63] reported that inhibition of STAT1 and STAT3 did not rescue cells from TNFα-induced senescence, led to increased p21 expression, and suppressed genes involved in interferon gene expression. 

These data provided clear evidence that STAT1 and STAT3 signaling activity is a feature of senescent preadipocytes: STAT1 is required to promote senescence growth arrest, and STAT3 promotes the survival of senescent preadipocytes. Our findings are consistent with what those performed in previous studies in different cell types have reported for the roles of STAT1 in inducing cell-cycle arrest and of STAT3 in enhancing cell survival [63,64]. It will be interesting to determine the changes associated with STAT3 deletion in terms of modulating the activation of apoptosis signaling in response to oxidative stress in senescent preadipocytes.

Next, we demonstrated a reduction in STING expression in STAT1 KO preadipocytes, which was reduced further in senescent preadipocytes compared to STAT3 KO preadipocytes. It is well known that the main outcome of the recognition of cytosolic dsDNA by the cGAS-STING signaling pathway is the induction of canonical type I interferon, including IFNα and IFNβ, that ultimately leads to the induction of interferon signaling-related genes and triggers STAT1 signaling [65]. Despite the knowledge that STAT1 is triggered following the initiation of cGAS-STING signaling, we showed here evidence for STING regulation by STAT1. It was recently reported that STAT1 could be activated in response to dsDNA and this response is not dependent on type I IFN receptors [66]. Further research will be required to determine the precise molecular mechanisms responsible for STING downregulation following STAT1 deletion in senescent preadipocytes.

Contrarily to STAT1, STAT3 deletion was associated with increased STING expression in proliferating preadipocytes accompanied by an increase in the expression of IFNα, IFNβ, CXCL10 and IL6. Consistent with our observations, emerging studies in different cell types reported that STAT3 deficiency contributed to enhanced antiviral responses and to increases in the expression of CXCL10 [67,68,69]. On the other hand, the link between STAT3 and IL6 has been reported in inflammation and cancer studies [70,71]. Therefore, understanding the molecular mechanisms associated with the functional interplay between STAT1 and STAT3 activation could provide a promising tool to control SASP secretion in senescent preadipocytes.

**Figure 9:**
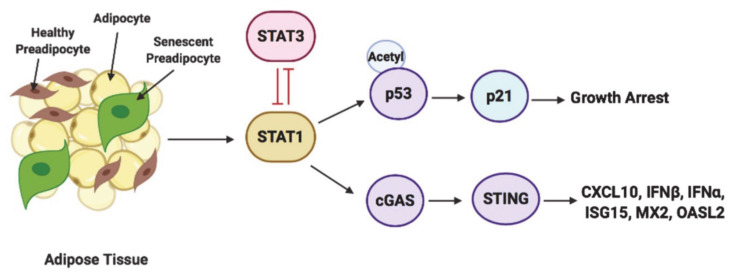
Model of STAT1 and STAT3 regulation of inflammation and growth arrest phenotypes in senescent preadipocytes. This schematic diagram represents how STAT1 and STAT3 function antagonistically in regulating cGAS-STING mediated induction of CXCL10 and interferon signaling related genes and p53/p21-dependent growth arrest. STAT1 cooperates with cGAS/STING in driving the induction of CXCL10, and interferon signaling related genes such as IFNα, IFNβ, ISG15, MX and OASL2 and antagonizes the function of STAT3 in inhibiting p53-dependent growth arrest and cGAS-dependent induction of interferon signaling related genes.

In summary, this study shows that STAT1 and STAT3 phosphorylation determine their antagonistic functions in regulating growth arrest and inflammation. We depict our findings in a model in Figure 9, where we report a critical function for the transcription factor STAT1 in mediating growth arrest and SASP characterized by the induction of interferon-related genes. Through this study, we also unveil a novel function for STAT3 in promoting cell survival through inhibiting acetylated p53/p21 and suppressing the activation of cGAS/STING, thereby inhibiting interferon signaling and reducing inflammation. These key findings suggest that targeting STAT1 and STAT3 signaling could be a therapeutic target for treating the inflammatory phenotype associated with senescence in obesity-driven T2DM.

## 5. Conclusions

In conclusion, our whole-transcriptome analysis-based discovery of disease-specific molecules and pathways provides a unique opportunity to specifically target disease mechanisms in vivo while having little/no impact on normal/healthy processes. This advantage will help in the identification of disease-specific circulating molecules that could be developed as diagnostic markers in obesity-driven T2DM.

## Data Availability

The data presented in this study are available within the article and in Supplementary Materials.

## Supplementary Materials

The following are available online at (https://www.mdpi.com/2076-3921/10/2/334/s1), Table S1: Table of primers used for gene expression analysis. Figure S1: Validation of upregulated SASP by dot blot. Figure S2: Graph for STAT1/3 protein quantification. Figure S3: ANOVA analysis for interferon signaling related genes in cGAS and STING knockout cells. Figure S4: Graph for protein Quantification. Figure S5A/B: Graph for protein quantification and Gene expression analysis for SASP molecules.
