# Supplementary material for: Signal Transducer and Activator of Transcription 3 (STAT3) Suppresses STAT1/Interferon Signaling Pathway and Inflammation in Senescent Preadipocytes
Source: Antioxidants (Basel). 2021 Feb 23;10(2):334. doi: 10.3390/antiox10020334 (PMC7927067; doi:10.3390/antiox10020334)
Supplement: Supplementary file 1 [file antioxidants-10-00334-s001.pdf]

antioxidants

SUPPLEMENTARY TABLES AND FIGURES

Table S1. Primers used for gene expression analysis in 3T3-L1, Stromal Vascular Fraction (SVF)-derived from mice and human samples.

Gene Symbol	Accession Number	Forward Primer (5'-3')	Reverse Primer (5'-3')
Mouse P53	NM_011640.3	GGCGTAAACGCTTCGAGATG	TTCAGGTAGCTGGAGTGAGC
Mouse P21	NM_007669.5	AGACATTCAGAGCCACAGGC	CGTCTCCGTGACGAAGTCAA
Mouse P16	NM_009877.2	TGGTCACTGTGAGGATTCAGC	TTGCCCATCATCATCACCTGG
Mouse TNFα	NM_013693.3	GGATGAGAAGTTCCCAAATGGC	GTTTGCTACGACGTGGGCTA
Mouse NFκB	NM_008689.2	AACTATGTGGGGCCTGCAAA	GTTTGCAAAGCCAACCACCA
Mouse IFNα	NM_010502.2	TTGATGGCAACCAGTTCCAG	TCATCCCAAGCAGCAGATGA
Mouse OAS1B	NM_001083925.1	GGGCCTCTAAAGGGGTCAAG	TCAAACTTCACTCCACAACGTC
Mouse STAT1	NM_001205313.1	CCTGGAGGTCTTTGTTCCCT	TGCGTTCAGACCTCTCTTGG
Mouse STAT3	NM_011486.5	ACCATTGACCTGCCGATGTC	ACGTGAGCGACTCAAACTGC
Mouse CXCL10	NM_021274.2	CCAAGTGCTGCCGTCATTTTC	GGCTCGCAGGGATGATTTCAA
Mouse MMP13	NM_008607.2	TGTTTGCAGAGCACTACTTGAA	CAGTCACCTCTAAGCCAAAGAAA
Mouse MMP3	NM_010809.2	ACATGGAGACTTTGTCCCTTTTG	TTGGCTGAGTGGTAGAGTCCC
Mouse IGFBP3	NM_008343.2	CCAGGAAACATCAGTGAGTCC	GGATGGAACTTGGAATCGGTCA
Mouse IGFBP4	NM_010517.4	AGAAGCCCCTGCGTACATTG	TTGTTGGGATGTTCGCTCTCA
Mouse IL6	NM_001314054.1	CTCTGCAAGAGACTTCCATCCA	ACAGGTCTGTTGGGAGTGGT
Mouse C3	NM_009778.3	CCAGCTCCCCATTAGCTCTG	GCACTTGCCTCTTTAGGAAGTC
Mouse CP	NM_001374677.1	GCACATGGGGTAACGTACACC	TCATCAGCCCGTTGAAAATCAG
Mouse OAS3	NM_145226.6	TTCGCAGAGCTTCGAAGGAA	TATATCGAGTGACAACCTGGCG
Mouse DPT	NM_019759.3	TGGATGGGTGAATCTTAACCGC	TCAGAGCCTTCCTTCTTGCTA
Mouse DCN	NM_001042651.1	AGCGAGAGGACTGCCATCTA	GTGGGTCTTTGTACCTGCTGT
Mouse IFNβ	NM_010510.1	CAGCTCCAAGAAAGGACGAAC	GGCAGTGTAACTCTTCTGCAT
Mouse ISG15	NM_015783.3	AGCAATGGCCTGGGACCTAA	TAAGACCGTCCTGGAGCACT
Mouse MX2	NM_013606.1	CACCAGGCTCCGAAAAGAGT	GGCAATTCTCGTCCACGGTA
Mouse OASL2	NM_011854.2	TGCCTGGGAGAGAATCGAGA	AGCCTCCCTTCACCACCTTA
Mouse RPLP0	NM_007475.5	AACCCTGAAGTGCTCGACAT	GAAGGCCTTGACCTTTTCAGT
Mouse B2M	NM_009735.3	GCTCACACTGAATTCACCCC	TGTCTCGATCCCAGTAGACG
Mouse CASP4	NM_007609.3	TCATGGCTGAAAACAAACACCC	AAACCCAACGCTTGTCACTG
Mouse IFI44	NM_133871.2	TGCACTCTTCTGAGCTGGTG	CCTCCAGCTTGGACTTCACA
Mouse CD74	NM_001042605.1	TACTGCTGGTGTGTGTTCCC	CAGGGTGACTTGACCCAGTT
Mouse XAF1	XM_006533567.3	ACCAGCAGACCAAGGAAAGC	ACTTGGAGTGTGATGGGCTG
Mouse RSAD2	NM_021384.4	CTGTGCGCTGGAAGGTTTTC	GCACCAAACAGGACACCTCT
Mouse USP18	NM_011909.2	ACACAGACTTGACAGAGCGG	TGAGCAGTTTGCTCCTCCTG

Human B2M	NM_004048.3	CACTGAATTCACCCCCACTGA	TTCAAACCTCCATGATGCTGC
Human OAS3	NM_006187.4	CAGAAGCCCAGGCCTATCAT	ACTTCACACAGCAGCCTTCA
Human CD74	NM_001025159.2	GATGCACCTGCTCCAGAATG	TTTTGCTCCAAGGAGTGCCT
Human CASP4	NM_001225.4	CCTATGGCAGAAGGCAACCA	TCTGCCATGACCCGAACTTT
Human USP18	NM_017414.4	GGCTCCTGAGGCAAATCTGT	AACCAGGCCATGAGGGTAGT
Human IFI44	NM_006417.5	ATTCCTCTGAGTGGGAGCTG	CCTCCCTTAGATTCCCTATTTGC
Human XAF1	NM_017523.5	AGCTCCACGAGTCCTACTGT	TTCTTTCCCCTTTCCCGAGC
Human Actin	NM_007393.3	TTCTACAATGAGCTGCGTGTG	GGGGTGTTGAAGGTCTCAAA

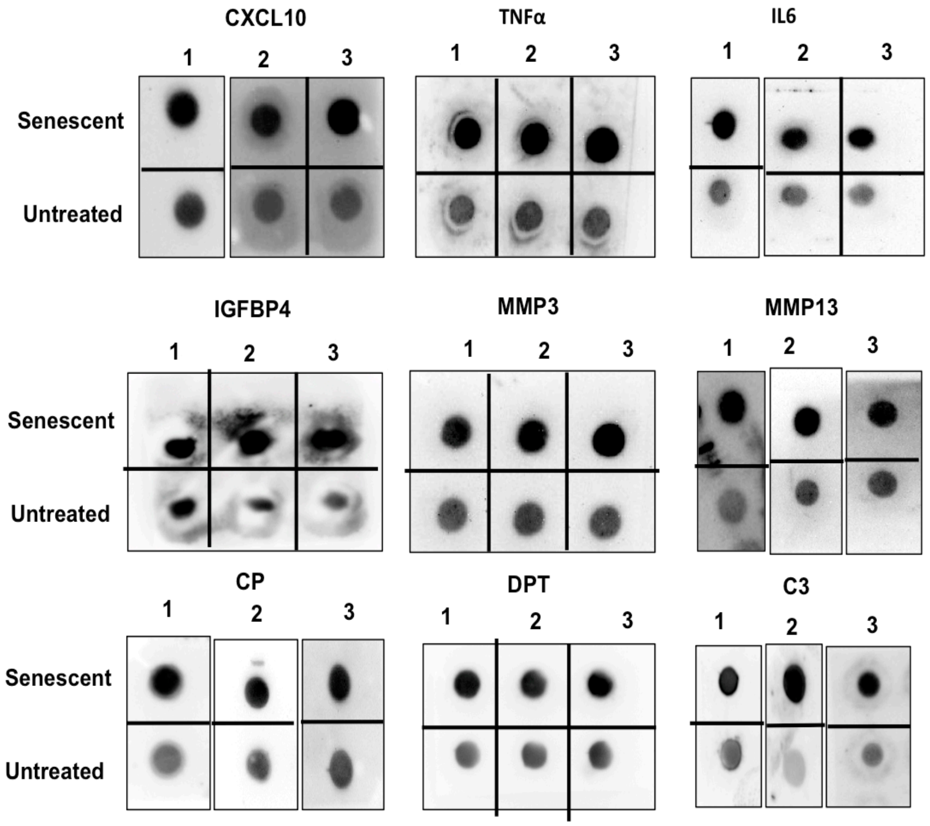

Figure S1. Validation of upregulated SASP by dot blot. Dot blot image used for evaluating the fold change in levels of the indicated SASP molecules in conditioned media of in senescent 3T3-L1 preadipocytes compared to untreated control cells. ImageJ quantification was performed for the intensity of the dot of the indicated molecule in conditioned media of senescent preadipocytes compared to untreated cells. Data are means ±SEM from 3 independent experiments. *$P \leq 0.05$ (Student's t-test).

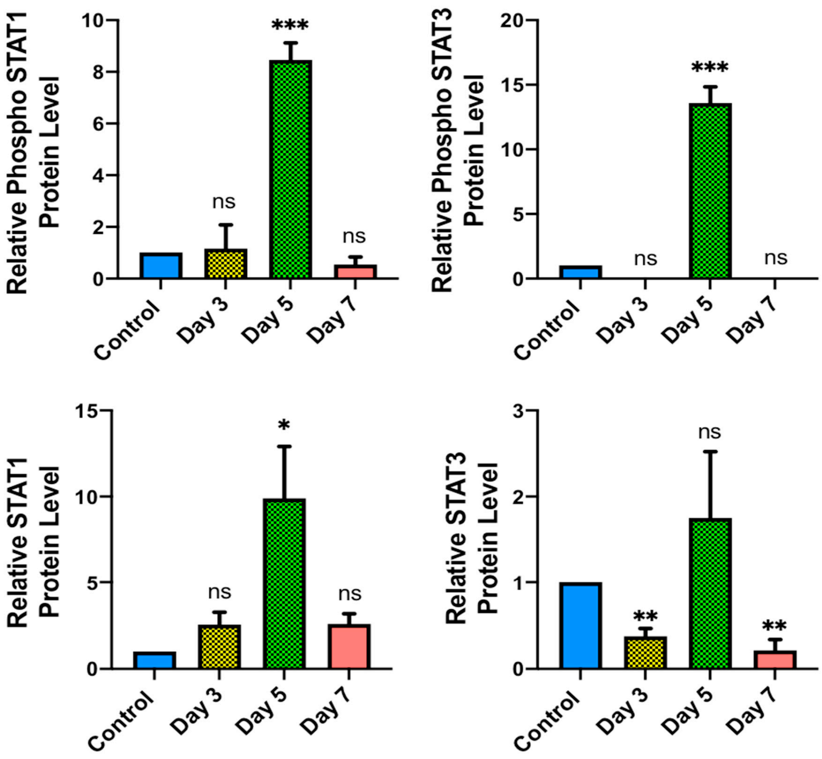

Figure S2. STAT1 and STAT3 activation in senescent preadipocytes. Graph showing quantified levels of each corresponding protein at different day 3, da5 and day7, which were normalized to those of actin. Results are represented as means ±SEM from three independent experiments. **P* ≤ 0.05, ***P* ≤ 0.01, ****P* ≤ 0.001 (Student's t-test).

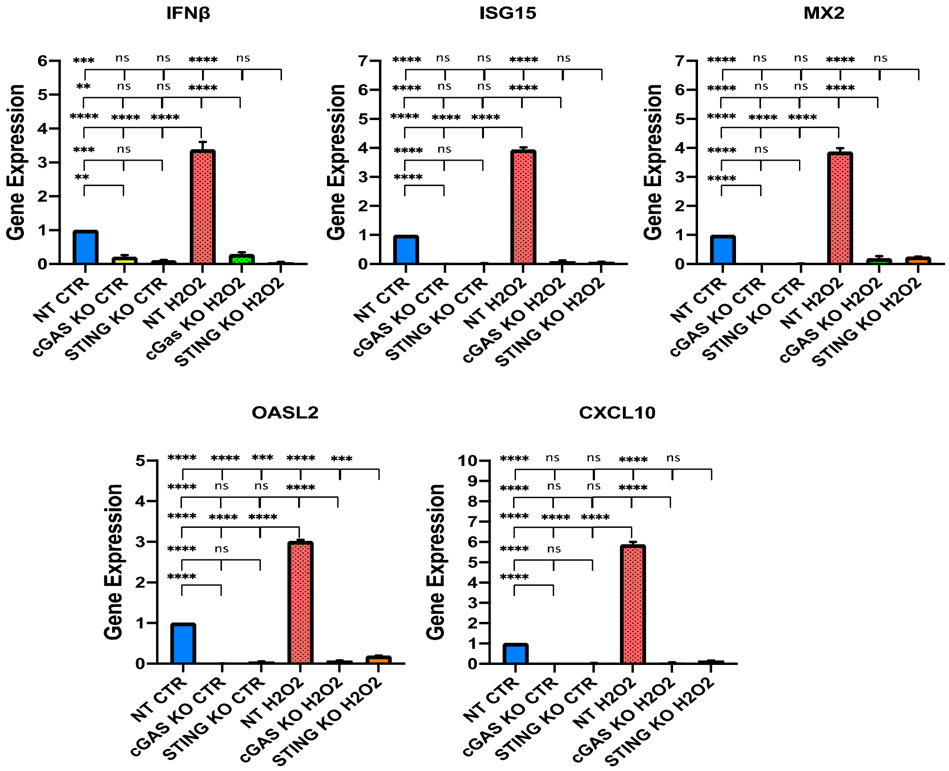

Figure S3. cGAS and STING regulates the gene expression of interferon signaling related genes in preadipocytes. Gene expression analysis for interferon signaling related genes (IFNβ, ISG15, MX2, OASL2 and CXCL10) in untreated NT, cGAS KO, and STING KO preadipocytes vs H_2O_2 treated counterparts as indicated. Results (Relative expression) are presented as means ±SEM from three independent experiments. $^{**}P \leq 0.01$, $^{***}P \leq 0.001$, $^{****}P \leq 0.0001$ (ANOVA with post-hoc Tukey test).

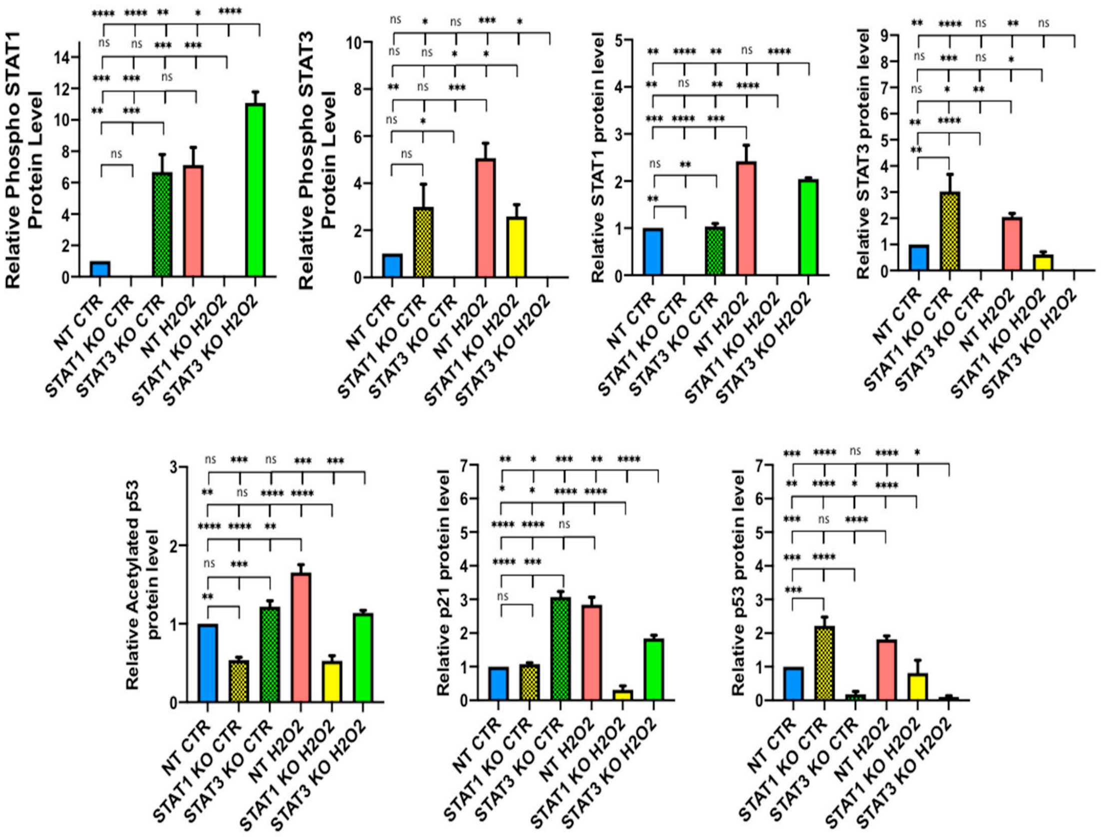

Figure S4. Antagonistic functions of STAT1 and STAT3 in regulating growth arrest and cell survival phenotypes in senescent preadipocytes. Graph showing quantified levels of each corresponding protein, which were normalized to those of actin. Results are represented as means ±SEM from three independent experiments. $^*P \leq 0.05$, $^{**}P \leq 0.01$, $^{***}P \leq 0.001$, $^{****}P \leq 0.0001$ (ANOVA with post-hoc Tukey test).

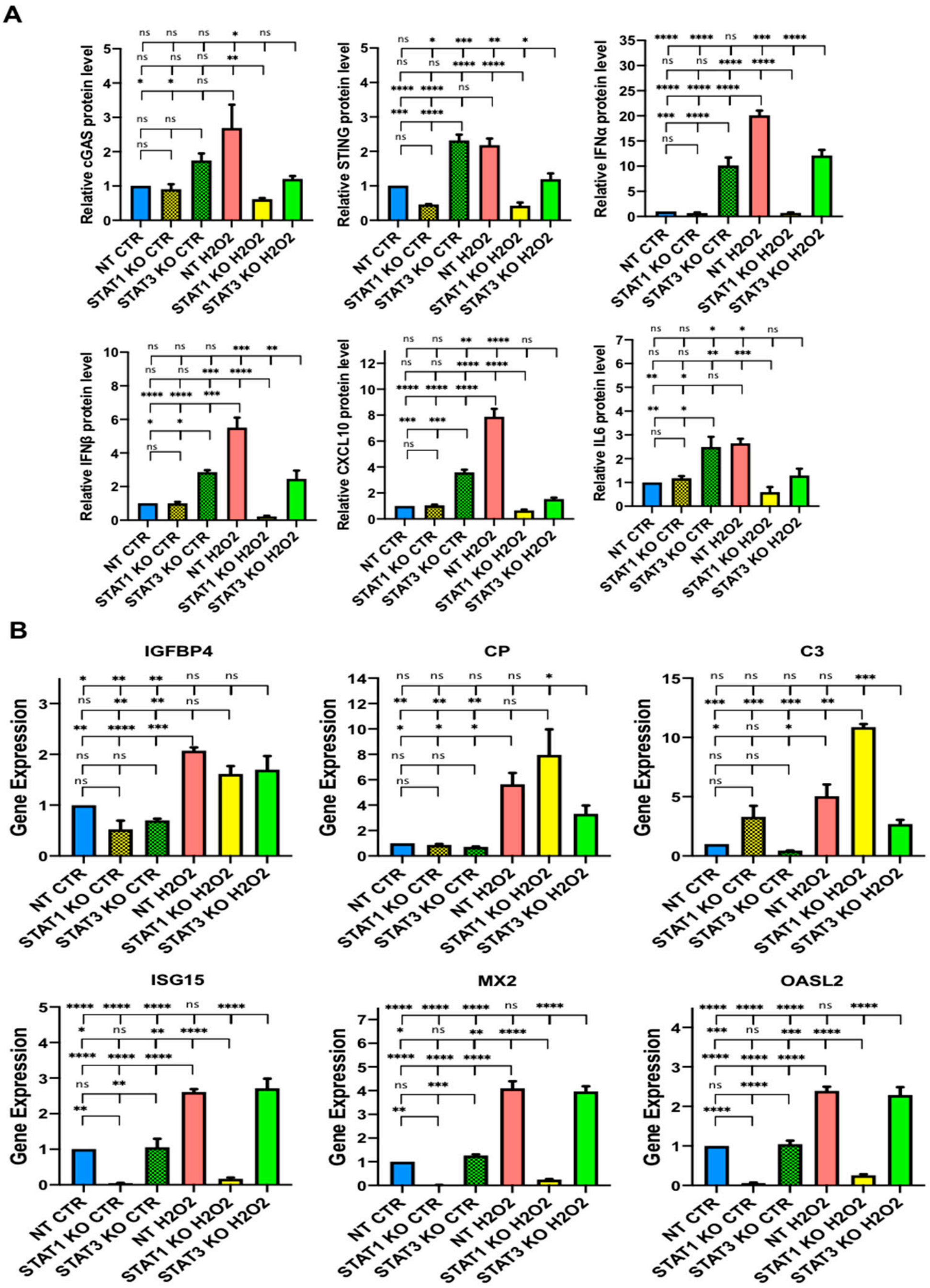

Figure S5. STAT1 functionally interacts with cGAS/STING to drive the expression of CXCL10 and antiviral response genes and STAT3 negatively regulates this interaction. (**A**) Graph showing quantified levels of each corresponding protein, which were normalized to those of actin. Results are represented as means ±SEM from three independent experiments. **P* ≤ 0.05, ***P* ≤ 0.01, ****P* ≤ 0.001, *****P* ≤ 0.0001 (ANOVA with post-hoc Tukey test). (**B**) Gene expression analysis of most significantly upregulated SASP molecules in untreated NT, STAT1 KO, and STAT3 KO preadipocytes vs H_2O_2 treated counterparts as indicated. Results (Relative expression) are presented as means ±SEM from three independent experiments. **P* ≤ 0.05, ***P* ≤ 0.01, ****P* ≤ 0.001, *****P* ≤ 0.0001 (ANOVA with post-hoc Tukey test).
